# Glucosylated cholesterol accumulates in atherosclerotic lesions and impacts macrophage immune response

**DOI:** 10.1016/j.jlr.2025.100825

**Published:** 2025-05-15

**Authors:** André R.A. Marques, Inês S. Ferreira, Quélia Ribeiro, Maria J. Ferraz, Elizeth Lopes, Daniela Pinto, Michael Hall, José Ramalho, Marta Artola, Manuel S. Almeida, Gustavo Rodrigues, Pedro Araújo Gonçalves, Jorge Ferreira, Cláudia Borbinha, João Pedro Marto, Miguel Viana-Baptista, Ryan Gouveia e Melo, Luís Mendes Pedro, Maria I.L. Soares, Winchil L.C. Vaz, Otília V. Vieira, Johannes M.F.G. Aerts

**Affiliations:** 1 iNOVA4Health, NOVA Medical School | Faculdade de Ciências Médicas, NMS | FCM, Universidade NOVA de Lisboa, Lisboa, Portugal; 2 Department of Medical Biochemistry, Leiden Institute of Chemistry, Leiden University, Leiden, The Netherlands; 3 Hospital Santa Cruz, Centro Hospitalar de Lisboa Ocidental, Carnaxide, Portugal; 4 Department of Neurology, Hospital de Egas Moniz, Centro Hospitalar de Lisboa Ocidental, Lisboa, Portugal; 5 Department of Vascular Surgery, Hospital de Santa Maria, Centro Hospitalar Universitário Lisboa Norte (CHULN), Lisboa, Portugal; 6 University of Coimbra, Coimbra Chemistry Centre - Institute of Molecular Sciences (CQC-IMS), Department of Chemistry, Coimbra, Portugal

**Keywords:** glucosylated cholesterol, glycosphingolipids, macrophage, multinucleated cells, lysosome

## Abstract

Atherosclerosis can be described as a local acquired lysosomal storage disorder (LSD), resulting from the build-up of undegraded material in lysosomes. Atherosclerotic foam cells accumulate cholesterol (Chol) and glycosphingolipids (GSLs) within lysosomes. This constitutes the ideal milieu for the formation of a side product of lysosomal storage: glucosylated cholesterol (GlcChol), previously found in several LSDs. Using LC-MS/MS, we demonstrated that GlcChol is abundant in atherosclerotic lesions. Patients suffering from cardiovascular diseases presented unaltered plasma GlcChol levels but slightly elevated GlcChol/Chol ratios. Furthermore, we mimicked GlcChol formation in vitro by exposing macrophages (Mφ) to a pro-atherogenic oxidized cholesteryl ester, an atherosclerosis foam cell model. Additionally, Mφ exposed to GlcChol exhibited an enlarged and multinucleated phenotype. These Mφ present signs of decreased proliferation and reduced pro-inflammatory capacity. Mechanistically, the process seems to be associated with activating the AMPK signaling pathway and the cyclin-dependent kinase inhibitor 1 (CDKN1A/p21), in response to DNA damage inflicted by reactive oxygen species (ROS). At the organelle level, exposure to GlcChol impacted the lysosomal compartment, resulting in the activation of the mTOR signaling pathway and lysosomal biogenesis mediated by the transcription factor EB (TFEB). This suggests that high concentrations of GlcChol impact cellular homeostasis. In contrast, under this threshold, GlcChol formation most likely represents a relatively innocuous compensatory mechanism to cope with Chol and GSL build-up within lesions. Our findings demonstrate that glycosidase-mediated lipid modifications may play a role in the etiology of genetic and acquired LSDs, warranting further investigation.

## Highlights

• Glucosylated cholesterol (GlcChol) is abundant in atherosclerotic lesions, identified via LC-MS/MS analysis.
• Cardiovascular disease patients showed unaltered plasma GlcChol levels but elevated GlcChol/Chol ratios.
• Macrophages exposed to oxidized cholesteryl ester mimicked GlcChol formation, reflecting atherosclerotic foam cell conditions.
• GlcChol exposure led to enlarged, multinucleated macrophages with decreased proliferation and reduced pro-inflammatory capacity.
• The phenotype is mechanistically connected with the induction of AMPK and CDKN1A/p21 activation in response to ROS-induced DNA damage.
• While elevated GlcChol levels can disturb cellular homeostasis, lower levels may act as a compensatory mechanism to manage cholesterol and glycosphingolipid accumulation.
• These findings suggest glycosidase-mediated lipid modifications may contribute to both genetic and acquired lysosomal storage disorders.

## Graphical Abstract

**Figure:**
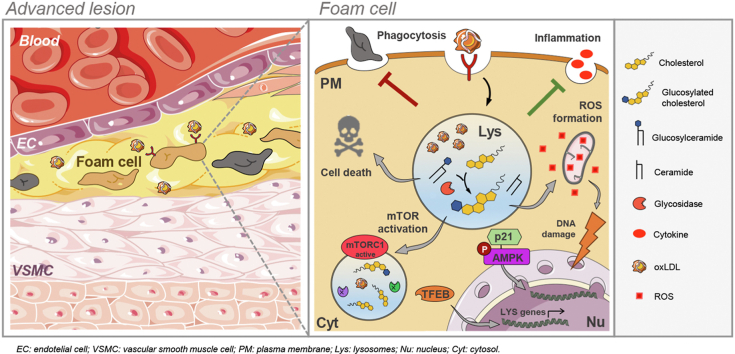

Atherosclerosis is a chronic inflammatory disease characterized by the deposition of lipids in the intima layer of large- and medium-sized arteries, especially at branch points and in areas of high vessel curvature. Atherosclerosis is the main underlying cause of cardiovascular diseases (CVDs), which account for almost half of all deaths related to non-communicable diseases, making them the single largest global cause of mortality (1).

According to the leading lipid hypothesis, elevated cholesterol (Chol) levels—hypercholesterolemia—constitute the major causative factor underlying plaque development (2). This hypothesis postulates that the retention of low-density lipoproteins (LDL) in the intima of arteries kick-starts a gradual pro-inflammatory cascade. Cholesterol esters (CE) are the main component of LDL (approximately 42% [wt/wt]), and so Chol metabolism plays a central role in the etiology of atherosclerosis (2). Once trapped in the intimal space, LDL can undergo modifications, such as aggregation and oxidation, that increase the atherogenicity of its components (2). We have previously shown that the end-products of the oxidation of LDL-derived CE, cholesteryl hemiesters (acylated cholesterol with a terminal carboxylic group), are highly atherogenic in *in vitro* plaque cell models (3, 4, 5).

Chol also undergoes metabolic conversion into metabolites with important functions as immune regulators in response to lipid-overload, such as oxygenated sterols (oxysterols), bile acids, and steroid hormones (6, 7). Recently, a new branch of Chol metabolism was discovered through the observation that glucosylated cholesterol (GlcChol) occurs in mammalian cells (8, 9). GlcChol can be formed and degraded by the action of two glycosidases, the lysosomal glucocerebrosidase (GBA) and the cytosol facing ER-resident GBA2 (8), previously thought to be exclusively involved in the catabolism of glycosphingolipids (GSLs) (8). Whenever donor (GlcCer) and acceptor (Chol) are available, both GBA and GBA2 can catalyze the transfer by transglucosylation of the glucose moiety from glucosylceramide (GlcCer) to the hydroxyl group of free Chol. Under normal conditions GBA2 is the main responsible for the synthesis of GlcChol, with its degradation occurring in the lysosome by GBA. However, under pathological conditions of lysosomal lipid accumulation, as observed in the LSDs Gaucher and Niemann Pick type C (NPC), the intralysosomal levels of GlcCer and Chol rise, enabling the formation of GlcChol by GBA (8). More recently, we have observed that GlcChol, as well as several plant glycosylated phytosterols, may also be acylated at the C6 of glucose. Furthermore, 6-*O*-acyl-glycosylcholesterols with different fatty acid chain lengths have been detected in human spleen samples. Interestingly, others have reported these lipids to be abundant in human atherosclerotic lesions (10).

The progressive lysosomal dysfunction in atherosclerotic plaque cells also constitutes a form of irreversible lipid accumulation, sharing many pathogenic pathways with LSDs (11). Lesions are distinctly rich in the GSLs GlcCer, lactosylceramide (LacCer), and ceramide (12, 13). These lipids have also been associated with several pro-atherogenic properties. Ceramide increases lipoprotein aggregation (14) and circulating ceramide levels are used as predictors of adverse cardiovascular events (15). GlcCer has been associated with plaque inflammation and instability (12). The *GBA* gene, underlying Gaucher disease, has been recently recognized as a risk factor for premature coronary heart disease (16). Patients with Gaucher disease also present reduced levels of high-density lipoprotein (HDL) cholesterol, yet this does not translate into increased CVD risk (17).

Little is known about the biological function of glycosylated sterols in mammals and the role of sterol glycosylation in atherosclerosis is yet to be addressed. Glycosylation will increase the amphiphilic character of sterols and thereby alter their mobility and hydrophobicity properties (18). In concrete, studies in glycerophospholipid and sphingolipid bilayers have demonstrated that cholesteryl glycosides partition more efficiently than Chol into lateral domains rich in phospholipids with disordered hydrocarbon chains (19). GlcChol was also shown to reduce sterol-sphingomyelin interactions, thereby possibly impacting the stability of membrane sphingomyelin-based domains (20). Chol glucosylation may be particularly important for the control of membrane microdomains in the central nervous system (CNS) (8, 20, 21, 22). Interestingly, GlcChol is enriched in extracellular vesicles associated with neurons and glial cells (22), pointing towards a potential role in central nervous system physiology (23). GlcChol has also been strongly linked to the regulation of the heat-shock response in mammalian cells. GlcChol production, upon heat shock, activates heat shock transcription factor-1 (HSF1) and leads to the production of heat shock protein 70 (HSP70) (23, 24).

The metabolism of Chol and its derivatives also plays a central role in the crosstalk between lipid-metabolic and immune-inflammatory atherogenic pathways (7). Cholesterol biosynthesis is directly regulated by innate immune responses, a fundamental process for proper macrophage (Mφ) function. For example, the downregulation of Chol biosynthetic genes in classically activated (pro-inflammatory) Mφ is linked to the regulation of inflammatory reactions and inflammasome suppression (25, 26, 27). The discovery of a new branch of sterol metabolism with the potential to deplete the pool of free sterols in plaque Mφ may strongly impact these regulatory mechanisms. Interestingly, exposure to GlcChol has been linked to apoptosis, via the induction of caspase-3 (28), and oxidative stress via reactive oxygen species (ROS) production (29). These observations are particularly relevant given the prominent roles played by apoptosis and oxidative stress in the etiology of atherosclerosis (30).

The lipid-laden lysosomes of atherosclerotic plaque cells provide the ideal environment for the formation of supraphysiological GlcChol. Here we set out to investigate whether GlcChol is enriched in human lesions, to confirm the molecular origin of this lipid, and obtain the first clues regarding its role in atherogenesis.

## Materials and Methods

### Chemicals

The activity-based probes ME569 (31) and JJB367 (32), ^13^C_6_-GlcChol (33) and GBA2 inhibitor N-(5-adamantane-1-ylmethoxy-pentyl)-deoxynojirimycin (MZ-21) (34) were synthesized in the department of Bio-organic Synthesis at the Faculty of Science, Leiden Institute of Chemistry at the University of Leiden as previously described. Cholesteryl hemiazelate (ChA) was synthesized at the Coimbra Chemistry Centre, University of Coimbra as previously described (3). The Rac1 inhibitor NSC23766 (#13196) was acquired from Cayman Europe. The remainder of the chemicals were acquired from Sigma-Aldrich.

### Synthesis of 1-*O*-cholesteryl-β-d-glucopyranoside (cholesteryl-β-d-glucoside)

*General Remarks:* Thin-layer chromatography (TLC) analyses were performed using precoated silica gel plates. Flash column chromatography was performed with silica gel 60 as the stationary phase. ^1^H NMR spectra were recorded on an instrument operating at 400 MHz. Chemical shifts are expressed in parts per million (ppm) relatively to internal tetramethylsilane (TMS) and coupling constants (*J*) are in hertz (Hz). Melting point was determined in open glass capillaries. Cholesterol, acetobromo-α-d-glucose, zinc oxide, and sodium methoxide were purchased from commercial sources and used as received. Toluene, chloroform, methanol, and dichloromethane were dried and purified by distillation. The detailed experimental procedure can be found in the Supplementary Information.

### Plasma and carotid endarterectomy specimens (CEA)

#### CEA specimens

Carotid atheroma plaques were isolated from patients with carotid artery disease (CAD) submitted to open endarterectomy at Hospital Santa Maria, Centro Hospitalar Universitário Lisboa Norte (CHULN) as described in detail in our previous publication (4) (Ref 209/18, de 27^th^ of July 2018).

#### Plasma samples

Details regarding the collection and processing of the patient’s plasma samples were previously published (35). The study was approved by the Ethical Review Board of the NMS of the NOVA University of Lisbon (n°06/2015/CEFCM).

All experiments were performed in accordance with the guidelines and regulations including, the Universal Declaration on Bioethics and Human Rights of UNESCO, 2005; The Charter of Fundamental rights of the EU, 2012; Ethical principles for medical research involving humans—Declaration of Helsinki, 2013; EU Regulation 2016/679 and Good Clinical Practice guidelines (Directive 2001/20/EC) and EU Clinical Trials Directive (2005/28/EC). Moreover, they complied with national legislation for the scientific use of human biological samples (Law N° 12/2005 and N° 131/2014).

### Murine aortic arches

Murine aortic arches from 3 adult Nur77-ApoE double knockout mice were a kind offer from Prof. Noam Zelcer (Department of Medical Biochemistry, Amsterdam UMC, Amsterdam Cardiovascular Sciences and Gastroenterology and Metabolism, University of Amsterdam).

### LDL sample

The LDL sample containing 2,232 mg/dl of ApoB was also a kind gift from Prof. Noam Zelcer.

### Lipid extraction, LC-MS/MS, data acquisition, and analysis

Determination of GSLs and lyso-GSLs from plasma, CEA, and cell lysates followed the protocol previously described (36). GlcChol quantitation followed the protocol established previously (33).

### Preparation of liposomes

1-Palmitoyl-2-oleoyl-glycero-3-phosphocholine (POPC, Avanti Polar Lipids) was used as a vehicle. POPC and ChA or cholesterol were mixed at a 35:65 M ratio. GlcChol:POPC liposomes were prepared with a molar ratio of 60:40. The detailed protocol for liposome preparation was described previously by our group (3). Sterol concentration was determined using the Liebermann protocol (37).

### Cell culture

RAW 264.7 cells (ATCC) were maintained and cultivated as described (5). Briefly, cells were grown in Dulbecco modified Eagle medium (DMEM Glutamax, Gibco, Thermo Fisher), supplemented with 10% heat inactivated fetal bovine serum (FBS, Gibco), 1% sodium pyruvate (Gibco), 1% PenStrep (Gibco). Cells were maintained at 37°C with 5% CO_2_ in a humidified incubator. Macrophages were then incubated with POPC, Chol:POPC, ChA:POPC or GlcChol:POPC liposomes for up to 72 h at different concentrations, as indicated in the figure legends. For the inhibition of the different enzymes involved in GlcChol metabolism, drugs were incubated for 72 h in simultaneous with liposomes as follows: GBA inhibitor Conduritol B epoxide (CBE, 500 μM), GBA2 inhibitor MZ-21 (20 nM), GCS inhibitor Eliglustat (100 nM) and LAL inhibitor Lalistat2 (30 μM). For treatment with methyl-β-cyclodextrin (CD), in the last 16 h of liposome treatment the media was removed and replaced with fresh media with 1 mM CD.

### MTS cell viability assay

Cell viability was measured using CellTiter 96 AQueous One Solution Cell Proliferation Assay (MTS, Promega, G3580). Cells were incubated for 1 h at 37°C 5% CO_2_ with 20 μl MTS, and the absorbance (490 nm) was assessed on a Synergy HT plate reader (BioTek) at 490 nm.

### Total and free cholesterol measurement

Measurement of total and free cholesterol from RAW 264.7 lysates as previously described (5).

### Staining, image acquisition, and analysis

Cells were grown on glass coverslips, treated, and fixed with 4% paraformaldehyde (PFA) for 20 min. Aldehyde groups were quenched with 10 mM ammonium chloride (NH_4_Cl). For TFEB and γH2Ax immunostaining, 0.2% Triton X-100 + 10% FBS in PBS was used to permeabilize the cells, followed by blocking with 0.1% Saponin + 10% FBS in PBS. For the remaining immunostainings, the permeabilization solution, 0.1% saponin in PBS, was used, followed by blocking with 1% BSA + 0.1% saponin in PBS. Cells were incubated overnight at 4 with primary antibodies (see supplemental Table S1). After washing 3 times with 0.1% saponin in PBS, coverslips were incubated with the secondary antibodies conjugated with Cy3 or Cy5 from Jackson ImmunoResearch Laboratories, DAPI (Fluka, 2491867) labeled the nuclei and Alexa fluor 488 phalloidin (Invitogen, A12379) or Alexa fluor 633 phalloidin (Molecular Probes, A22284) labeled F-actin. Coverslips were washed 3 times with 0.1% saponin in PBS and twice with Milli-Q® water and mounted with Dako Fluorescence Mounting Medium (Dako, S3023). Images were obtained using an LSM980 Confocal system with Airyscan with a 63x oil-immersion 1.4 NA objective.

### Image analysis and lysosome quantification

Quantification analysis of lysosomes was performed using CellProfiler software (version 4.2.6) through a customized pipeline. Initially, the Threshold module was applied for background correction and intensity normalization. The images were then smoothed with the Smooth module, which can be helpful to remove small artifacts. Next, lysosome and cell identification were performed based on fluorescence intensity using the *IdentifyPrimaryObjects* module, with parameters adjusted to optimize the detection. The calculation of lysosomes per cell number was achieved with *RelateObjects* module. Lastly, the *MeasureObjectSizeShape* module was applied for quantification of geometric parameters (i.e., area) of lysosomes and lysosomes per cell.

### Western blotting

Cell lysates were prepared using RIPA lysis buffer with protease (cOmplete, EDTA-free, 1187580001) and phosphatase (Calbiochem, 524625) inhibitors, as previously described (3, 5). Protein concentration was determined using the Pierce™ BCA Protein Assay Kit (Thermo Scientific, 23225). Samples were then mixed with sample buffer, heated for 5 min at 95°C, and 20–50 μg were loaded into a 10, 12 or 15% SDS polyacrylamide gel. After electrophoresis, proteins were transferred to an activated PVDF or nitrocellulose membrane in transfer buffer at 4°C for 1 h. Membranes were blocked with blocking buffer (5% BSA or milk in TBS-T–Tris-buffered saline, 0.01% tween) for 1 h at room temperature (RT), followed by overnight (ON) incubation at 4°C with the primary antibodies (see supplemental Table S1) diluted in blocking buffer. After incubation, membranes were washed with 1X TBS-T and incubated for 2 h at RT with the corresponding horseradish peroxidase secondary antibody (Bio-Rad Laboratories) diluted in blocking buffer. Detection was performed using Clarity™ ECL substrate (Bio-Rad laboratories, 170–5061) in a Chemidoc Touch Imaging System (Bio-Rad Laboratories). Bands were quantified using the Fiji software, v1.53f51.

### Quantitative RT-PCR

The total RNA was extracted with the NZY total RNA isolation kit (Nzytech, MB13402) and reverse transcription was performed using NZY first-strand cDNA synthesis kit (Nzytech, MB12501) following the manufacturer's instructions. Quantitative PCR was performed in a 96-well plate using NZYSupreme qPCR Green Master Mix (Nzytech, MB44003) in a QuantStudio™ 5 Real-Time PCR System (Thermo Fisher). GAPDH and PGK1 were used as housekeeping genes to normalize gene expression. Target gene expression was determined by relative quantification (2^-ΔΔCt^ method) to the housekeeping reference gene. The primers were ordered from Thermo Fisher, and the sequences are indicated in Table 1.

**Table 1:** Primers sequences for RT-PCR

Genes	Sequences	
	Forward	Reverse
*Mcln1*	GCGCCTATGACACCATCAA	TATCCTGGCACTGCTCGAT
*Mki67*	AATCCAACTCAAGTAAACGGGG	TTGGCTTGCTTCCATCCTCA
*Tfeb*	AGGAGCGGCAGAAGAAAGAC	CAGGTCCTTCTGCATCCTCC
*Pgk1*	ATGGATGAGGTGGTGAAAGC	CAGTGCTCACATGGCTGACT
*Gapdh*	GGGAAGCCCATCACCATCTTC	AGAGGGGCCATCCACAGTCT

### Apoptosis assay

RAW264.7 cells, after 72 h incubation with the respective lipids, were first washed twice, on ice, with 1 ml of cold FACS buffer (PBS with 1% FBS). Cells were then detached by gentle scraping and transferred to pre-chilled Eppendorf tubes. The cell suspension was centrifuged at 300 *g* for 5 min at 4°C, and the supernatant was discarded. The staining was performed using the FITC Annexin V Apoptosis Detection Kit with PI (Biolegend, 640,914), by resuspending the pellet in 300 μl of Annexin Binding Buffer and adding to each tube, 1 μl of FITC-conjugated Annexin V and 3 μl of propidium iodide (PI). The samples were gently vortexed and incubated at RT for 15 min in the dark. Stained cells were transferred to 5 ml FACS tubes and analyzed by flow cytometry using the BD FACSCanto II. The results were processed using FlowJo™ v10.10.

### Cell cycle assay

Cells were first washed twice with 1 ml of PBS. Cells were detached and collected by gentle scraping and transferred to pre-chilled Eppendorf tubes. The cell suspension was centrifuged at 300 *g* for 5 min at 4°C, and the supernatant was discarded. The cell pellet was resuspended dropwise in 1 ml of ice-cold 70% ethanol while vortexing. Cells were incubated on ice for 30 min. Following fixation, cells were washed and then resuspended in 50 μl of RNase solution (100 μg/ml) and 400 μl of PI solution (50 μg/ml in PBS). The suspension was gently vortexed and incubated at RT for 30 min in the dark. The stained cells were then transferred to 5 ml flow cytometry tubes and analyzed using flow cytometry (BD FACSCanto II) with appropriate machine settings to determine cell cycle distribution. The results were processed using the Watson pragmatic fitting algorithm in FlowJo™ v10.10.

### Lysosome pH assay

RAW 264.7 cells were cultured in a LabTek chamber for 72 h. Lysosomal pH was evaluated following an ON incubation with 50 μg/ml of pHrodo Green Dextran conjugate (Invitrogen, P35368) and 50 μg/ml of Alexa Fluor™ 647 conjugated dextran (Invitrogen, D22914). After incubation, the dextran-containing medium was discarded, and the cells were rinsed with DMEM without phenol red (Gibco, 21063-029). Live imaging was carried out with cells maintained in HEPES-buffered HBSS at 37°C. Images were acquired using a Zeiss LSM980 confocal microscope equipped with a 63x oil-immersion objective (1.4 NA) and a microincubator set to 37°C. The fluorescence intensity ratio between pHrodo- and Alexa Fluor 647-dextran was determined using ImageJ Fiji software. Lysosomes were identified using the Threshold function, and, after background subtraction, fluorescence intensity from both channels was quantified using the Coloc2 plugin.

To determine absolute pH values, cells were sequentially exposed to isotonic K^+^ solutions adjusted to pH values between 4.0 and 7.0. This calibration was performed in the presence of 10 μM nigericin and 5 μM monensin, following previously established protocols (38).

### Electron microscopy with BSA-Gold

Cells were seeded on glass coverslips, and following 72 h of incubation, a 2-h pulse with BSA-Gold was performed. Preparation of BSA-gold conjugate was performed as previously described (39). After the pulse, BSA-Gold media was removed and media with liposomes was added once again for a chase of 4 h. Next, cells were fixed with 2% glutaraldehyde (GA) and 2% PFA in 0.1 M sodium phosphate buffer (PB) at pH 7.4 buffer ON at 4°C. The glass coverslips were washed 2X with 0.05 M PB with 0.1 M PB, and stored in 0.1 M PB at 4°C. Specimens were postfixed for 1 h on ice with 1% osmium tetroxide and 1.5% potassium ferrocyanide and then incubated with 1% tannic acid for 30 min at RT before dehydrating with ethanol and infiltrating/embedding on Epon resin-filled BEEM capsules (EMbed 812). After polymerizing the resin at 65°C ON, the glass and BEEM capsules were separated by dipping into liquid nitrogen, blocks were sectioned at 70 nm thickness using a Leica UC7 ultramicrotome with a diamond knife (Diatome), and sections were collected on formvar and carbon-coated copper slot grids. Sections were post-stained with 2% uranyl acetate in 70% methanol, followed by Reynold’s lead citrate and imaged with a Hitachi H-7650 TEM equipped with an AMT XR41 M digital camera. The embedding and imaging were performed at the Electron Microscopy Facility at the Gulbenkian Institute for Molecular Medicine.

### Phagocytosis assay

IgG-opsonized latex beads (3.87 μm) were prepared as described before (40). The cells were pulsed with IgG-opsonized particles for 30 min. After washing with PBS, the cells were fixed with 4% PFA and the non-internalized beads were immunostained with Cy5-conjugated donkey anti-human IgG antibody (709-175-149, Jackson Immuno Research) and DAPI.

### Fluorometric ROS assay

The cells were seeded on a black 96-well plate with a clear bottom (Nunc) and exposed to the liposomes for 72 h. Afterwards, the media was replaced with DMEM without phenol red supplemented with sodium pyruvate and 20 mM of N-acetyl-cysteine were added to the indicated wells and incubated for 30 min. Then, 2.5 mM of positive control H_2_O_2_ was added and incubated for an additional 2 h. Afterward, the cells were stained with 5 μM of the CellRox Green probe (C10444, Invitrogen) for 30 min, according to the manufacturer’s instructions. The cells were maintained in HEPES-buffered HBSS and fluorescence (λex 505, λem 530) was read on SpectraMax i3x Multi-Mode Microplate Reader (Molecular Devices). The protein content of each well was then determined using the Pierce™ BCA Protein Assay Kit for normalization of the fluorescence signal.

### Multiplex analysis of cytokines

Supernatants from RAW cultures stimulated with lipids for 24 h, followed by an additional 24 h incubation with LPS, were collected and stored at −20°C. IL-1α, TNF-α, CCL2 (MCP-1), and IL-6 levels in conditioned supernatants were determined using a multiplex bead array kit by BioLegend, which was used according to the provided manual (LEGENDplexTM Mouse Inflammation Panel (13-plex) with V-bottom Plate; # 740446). The assay was performed on 25 μl of diluted conditioned supernatants (2× or 5×). Samples were read on the BD FLOW FACS Canto II flow cytometer platform. Data was analyzed with BioLegend’s LEGENDplexTM Data Analysis Software.

### Statistical analysis

Statistical significance was assessed using GraphPad software by one-way ANOVA with Tukey post-test, Kruskal–Wallis with Dunn's multiple comparisons test, two-way ANOVA with Tukey post-test or Student's *t* test, depending on data distribution. A *P* value of 0.05 was considered to be statistically significant.

## Results

### GlcChol accumulates in the core of human atherosclerotic lesions

**Fig. 1:**
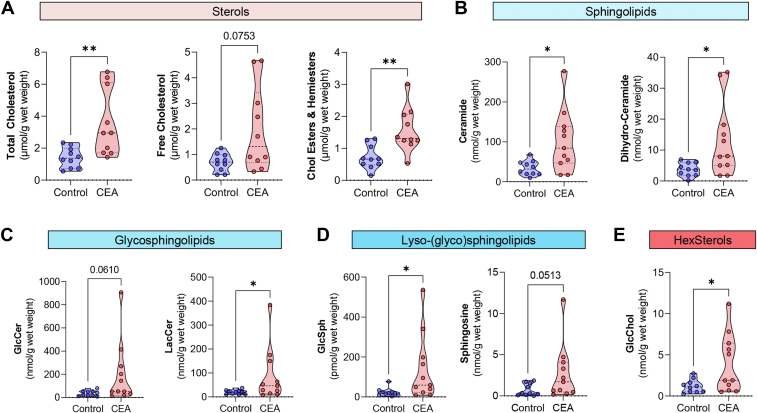
Glycosphingolipids and glucosylated-cholesterol accumulate in the core of human atherosclerotic lesions. Lipid levels in human CEA specimens and control (surrounding) tissue determined by LC-MS/MS or fluorescent enzymatic assay (cholesterol). A: Levels of total cholesterol, free cholesterol, and cholesteryl esters and hemiesters (indistinguishable with this technique) (micromole per gram of wet weight). B: Levels of the sphingolipids ceramide and dihydro-ceramide (nanomole per gram of wet weight) in human CEA samples. C: Levels of the glycosphingolipids glucosylceramide (GlcCer) and lactosylceramide (LacCer) in CAE samples (nanomole per gram of wet weight). D: Lyso-glycosphingolipid glucosylsphingosine (GlcSph, picomole per gram of wet weight) and sphingosine (nanomole per gram of wet weight) in CEA samples. (E) Hexosylated sterol (HexSterol) – glucosylated-cholesterol levels (GlcChol, nanomole per gram of wet weight), confirmed by HILIC chromatography. Data = violin blot of 6–11 samples. The *P* values were obtained by Mann-Whitney test; ∗∗*P* < 0.01; ∗*P* < 0.05.

Considering that both precursors of cholesterol (Chol) transglucosylation, GlcCer and Chol, are known to accumulate in atherosclerotic lesions and to play a role in the aetiology of the disease (12), we first set out to determine whether GlcChol is present in human plaques. Human lesions were collected from patients subjected to carotid endarterectomy (CEA). The samples were dissected into maximally diseased atherosclerotic regions and surrounding tissue, used as controls. We then proceeded to analyze the glycolipid and Chol composition of these specimens by established LC-MS/MS methods with ^13^C-labeled internal standards (33, 36) and enzymatic assays (5), respectively. As expected, lesions were enriched in total Chol levels (*P* < 0.01, Fig. 1A), due to a significant enrichment in Chol esters (*P* < 0.01, Fig. 1A). Free Chol levels were not significantly different between control and diseased tissues (*P* = 0.075, Fig. 1A). Several studies have previously described an enrichment in sphingolipids and GSL in plaques (12). Accordingly, we observed significant increases in several sphingolipid species, such as ceramides and dihydro-ceramides in CEA compared to control tissue (*P* < 0.05, Fig. 1B). We also observed an increase, just short of significance, in the GSLs GlcCer (*P* = 0.06, Fig. 1C) and LacCer (*P* < 0.05, Fig. 1C). In the field of LSD research, the deacylated sphingoid bases of the main accumulated GSLs are often employed as disease biomarkers rather than the respective GSL (41). LysoGSLs are formed through a deacylation reaction catalyzed by the enzyme acid ceramidase (42). The CEA specimens examined were enriched in all the species quantified – sphingosine (*P* = 0.051), glucosylsphingosine (*P* < 0.05), lyso-sphingomyelin (*P* < 0.05), lactosylsphingosine (*P* < 0.05), and lysoGb3 (*P* = 0.05); except sphinganine (Fig. 1D and supplemental Fig. S1). Importantly, GlcChol was significantly (*P* < 0.05, Fig. 1E) elevated in the atherosclerotic lesions compared to the surrounding vessel material. GlcChol levels were in the range of 0.2–2.7 nmol per gram wet weight in control samples and 0.5–11.2 nmol per gram wet weight in CEA specimens, an order of magnitude similar to the sphingoid base sphingosine and the GSL dihydro-ceramide. Additionally, we observed that GlcChol is also relatively abundant in aortic arches of a murine atherosclerosis model (Nur77-ApoE double knockout mice, range: 27–314 pmol per gram wet weight, n = 3). LC-MS/MS analysis of the human and murine specimens employing an HILIC (hydrophilic interaction liquid chromatography) column, which enables the separation of galactosylated from glucosylated Chol, revealed that >99% of the glycosylated sterol present in these samples is indeed GlcChol and not GalChol (data not shown). We also tested the activity of several lysosomal hydrolases but found no significant differences between control tissue and CEA (supplemental Fig. S1).

### The ratio of GlcChol to total cholesterol is elevated in patients who suffered myocardial infarction

**Fig. 2:**
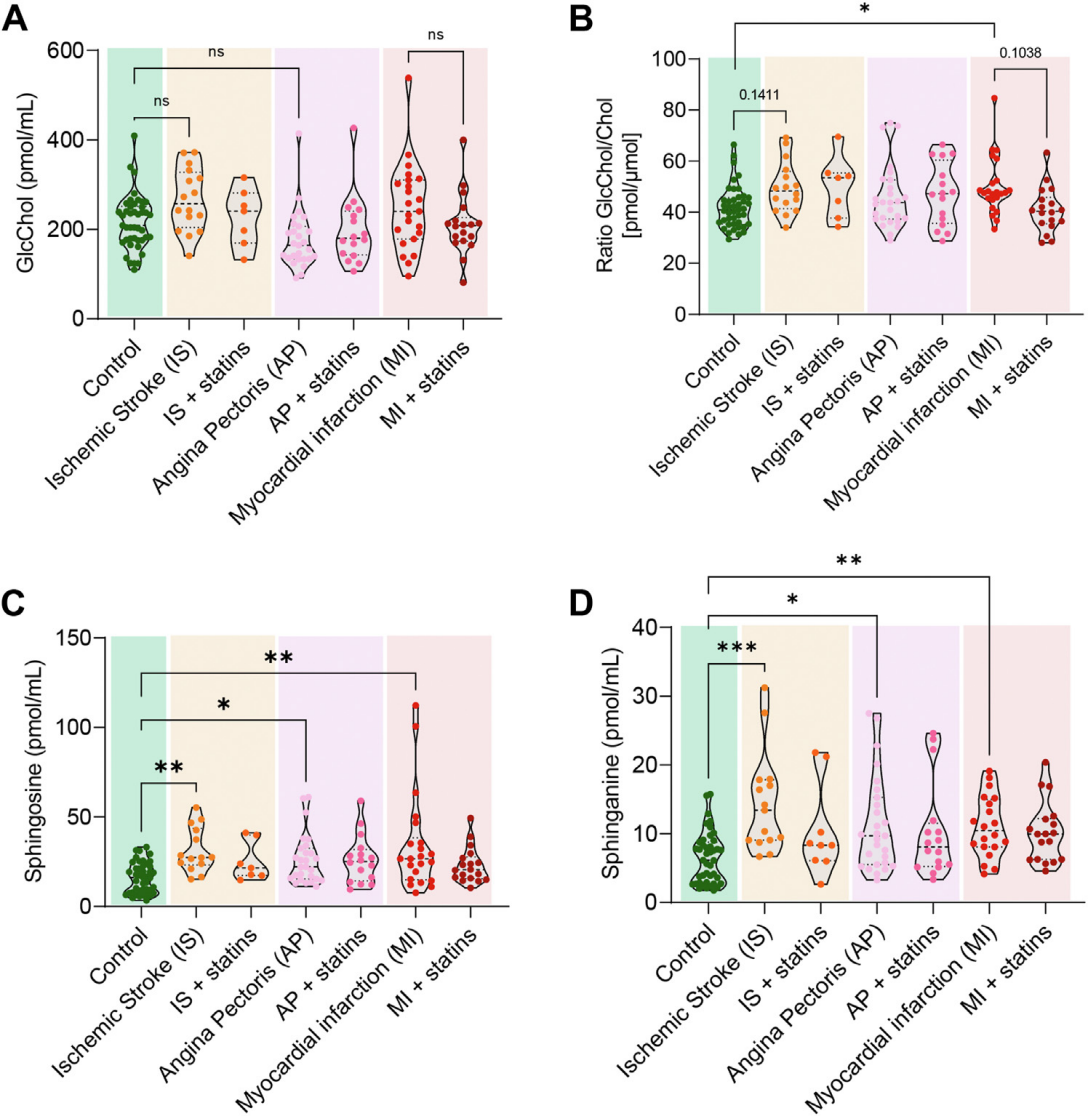
The ratio glucosylated/total cholesterol is increased in the plasma of patients that suffered myocardial infarction. A: GlcChol (picomole per millilitre) levels, B: glucosylated/total cholesterol ratio (picomole per micromole), C: sphingosine, and D: sphinganine levels (picomole per millilitre) in the plasma of patients that suffered from ischemic stroke, angina pectoris and myocardial infarction, and healthy age- and gender-matched individuals (N = 22–40). Patients were divided according to the prescription or not of statins at the date of sample collection. Data mean ± SD analyzed by Kruskal-Wallis followed by Dunn’s multiple comparison test; ns = nonsignificant; ∗*P* < 0.05, ∗∗*P* < 0.01, ∗∗∗*P* < 0.001.

Next, we wanted to investigate whether this local elevation of GlcChol levels in plaques would be reflected in the circulation of patients who suffered a cardiovascular event connected to atherosclerosis development. We assessed glycolipid levels in the plasma of patients who had suffered an ischemic stroke, angina pectoris, or myocardial infarction. The blood was collected upon hospitalization, and the cohorts were separated according to whether the patients were receiving statin treatment at that moment. Although circulatory GlcChol levels were not significantly altered in any of the CVD cohorts compared to the control group (Fig. 2A), upon normalization to total cholesterol levels, to account for the differences in lipoproteins in the circulation, we did observe a significant increase in the ratio GlcChol/Chol in the myocardial infarction cohort (*P* < 0.05, Fig. 2B). This effect was absent in the cohort of patients undergoing statin treatment at the time of the event.

In accordance with previous studies, inflammatory lipid mediator sphingosine and sphinganine levels were significantly elevated in the 3 CVD cohorts compared to the control cohort (Fig. 2C, D). Again, patients treated with statins did not present elevations in the plasma levels of these two sphingoid bases when compared to controls (Fig. 2C, D). Other GSLs and lyso-GSLs were unaltered in the plasma of the CVD cohorts, except for a significant reduction in Lyso-Gb3 in patients suffering from myocardial infarction (supplemental Fig. S2).

To confirm whether GlcChol is associated with lipoproteins in the circulation, we determined its levels in LDL isolated from one healthy individual. The LDL sample contained 1.63 nmol/ml (0.0896 mg/dl) of GlcChol and 40.6 nmol/ml (2,332 mg/dl) of ApoB, evidencing a relative abundance of this lipid in lipoproteins of circa one GlcChol molecule per 25 ApoB100 molecules, i.e., LDL lipoproteins. Furthermore, taking into consideration an LDL-cholesterol range between 85 and 130 mg/dl (2.2–3.4 μmol/ml) for a healthy individual, we can estimate that GlcChol should represent between 0.05% and 0.07% of total cholesterol levels in LDL.

### GlcChol accumulates in an atherosclerosis macrophage foam cell model

Having established that GlcChol is an abundant lipid component of human and murine atherosclerotic lesions, we next set out to investigate whether we could mimic its accumulation in *in vitro* foam cell models. Plaque foam cells can be derived from macrophages (Mφ) or vascular smooth muscle cells (VSMCs) that become lipidotic due to the unrestrained uptake of oxidized LDL by scavenger receptors (30). We have previously shown that a single end-product of the oxidation of (LDL-derived) cholesteryl esters, cholesteryl hemiazelate (ChA), can reproduce the conversion of Mφ and VSMCs into foam cells *in vitro*, preserving the distinct features of these two populations (3, 5).

**Fig. 3:**
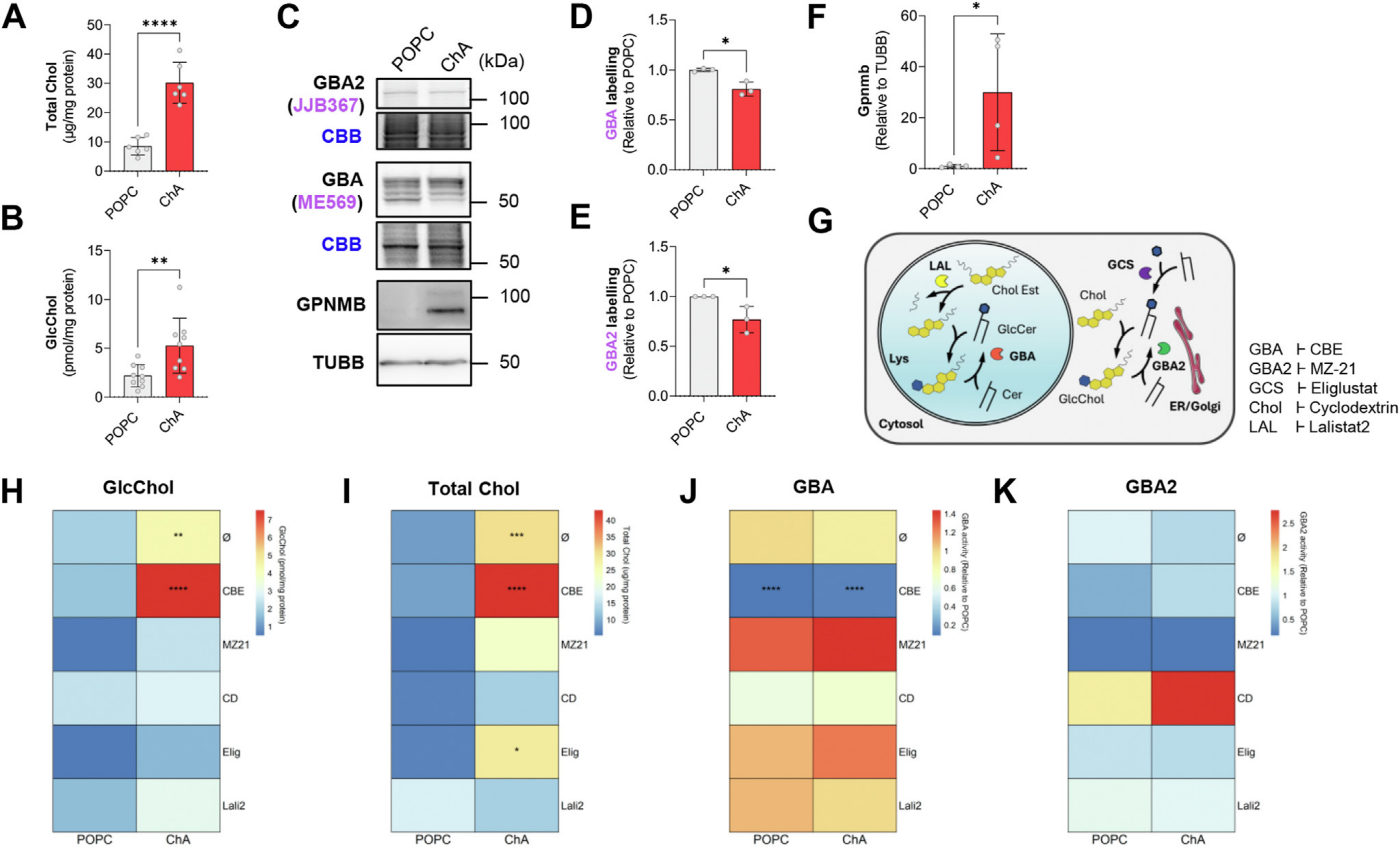
Macrophages exposed to cholesteryl hemiazelate (foam cell model) accumulate GlcChol. A: Fluorometric quantification of total cholesterol (microgram per milligram of protein) in lysates of macrophages exposed for 72 h to 807 μM POPC (vehicle) or 1500 μM cholesteryl hemiazelate (ChA). The results are mean ± SD of 6 independent experiments. The *P* values were obtained by unpaired *t* test; ∗∗∗∗*P* < 0.0001. B: Levels of GlcChol determined by LC-MS/MS (picomole per milligram of protein) in lysates of RAW 264.7 cells exposed to ChA or POPC for 72 h. The results are mean ± SD of 6 independent experiments. The *P* values were obtained by unpaired *t* test; ∗∗*P* < 0.01. C: Fluorescent labeling and quantification of active GBA with the ABP ME569 (D) and GBA2 with the ABP JJB367 (E) in lysates of cells exposed to POPC or ChA for 72 h. Gels were stained with Coomassie Brilliant Blue (CBB) as loading control. Data = mean ± SD of 3 independent experiments, ∗*P* < 0.05 (unpaired *t* test). Immunoblot (C) and densiometric quantification of the protein levels of gpNMB (F) in lysates of cells treated with POPC (vehicle) or ChA for 72 h. Protein levels were normalized to tubulin (TUBB) levels. Data = mean ± SD of 4 independent experiments; ns = nonsignificant; ∗*P* < 0.05 (unpaired *t* test). G: Schematic of the cellular metabolism of GlcChol. Under physiological conditions the glucosidase GBA2 localized in the ER/Golgi catalyzes the formation of GlcChol using free cholesterol (Chol) and the glucose from glucosylceramide (GlcCer) as substrates. GlcCer is also formed in the Golgi. Under these conditions GlcChol is degraded in the lysosome through the action of the glucosidase GBA, yielding free Chol and glucose. Cholesteryl esters and hemiesters are degraded in the lysosome by the lysosomal acid lipase (LAL), causing the release of free Chol, which may in turn be used for the synthesis of GlcChol taking place in the lysosome under conditions of local Chol accumulation. Heatmaps representing GlcChol levels (picomole per milligram of protein) (H), total Chol levels (microgram per milligram of protein) (I), GBA activity (relative to POPC) (J) and GBA2 activity (relative to POPC) (K) in RAW 264.7 cells exposed to POPC or ChA for 72 h in simultaneous with CBE (GBA inhibitor), MZ21 (GBA2 inhibitor), cyclodextrin (CD) (lysosomal cholesterol exporter), Eliglustat (GCS inhibitor) or Lalistat2 (LAL inhibitor). Data = mean ± SD of 3–12 independent experiments, ∗*P* < 0.05, ∗∗*P* < 0.01, ∗∗∗*P* < 0.001, ∗∗∗∗*P* < 0.0001 (ANOVA-Tukey test relative to control POPC).

Here, we have employed these models to study GlcChol formation *in vitro*. In the murine VSMC model, after exposure to ChA, despite a significant increase in total cellular Chol levels, we did not observe an alteration in GlcChol levels compared to the control cells (supplemental Fig. S3). As we previously reported, exposure of the murine Mφ-like cell line RAW 264.7 to ChA liposomes for 72 h results in a substantial (3-fold) increase in cholesterol levels, compared to vehicle treated cells (Fig. 3A). Despite there not being a significant elevation in the levels of the glucose donor GlcCer (supplemental Fig. S3) needed for the transglucosylation reaction (8), the levels of GlcChol were 4-fold increased (*P* < 0.01) in cells exposed to this oxidized CE (Fig. 3B). This indicates that the accumulation of Chol, likely in the lysosomal compartment, was sufficient to drive GlcChol formation.

GlcChol metabolism is dynamic and dependent on the local availability of substrates for the transglucosylation reaction mediated by glucosidases GBA and GBA2 (Fig. 3G). Interestingly, the activity levels of both the lysosomal GBA and the cytosol-facing GBA2 glucosidases were directly affected by treatment of ChA, presenting a significant reduction in both cases (Fig. 3C–E). The lysosome stress marker glycoprotein NMB (gpNMB), also associated with impaired GBA activity in LSDs (43, 44), is dramatically increased in Mφ exposed to ChA (Fig. 3F). Overall, Mφ-derived foam cells seem to be more prone to accumulate GlcChol than VSMC-derived foam cells.

Next, to investigate the origin of the GlcChol accumulated in ChA-treated Mφ, we pharmacologically inhibited several key enzymes for GlcChol metabolism, namely GlcCer synthase (GCS), GBA, GBA2, and LAL (Fig. 3G). GCS is a (Golgi) membrane-anchored enzyme that catalyzes the synthesis of GlcCer from ceramide and UDP-glucose. We also promoted the extraction of lysosomal Chol by treatment with methyl-β-cyclodextrin (CD). In both control and ChA-treated cells, inhibition of GBA2, LAL, and GCS, and CD treatment resulted in a decrease in GlcChol levels (Fig. 3H), confirming the dependency of the activity of these enzymes. In contrast, inhibition of GBA exacerbated the GlcChol build-up in ChA-treated Mφ but not in control cells (Fig. 3H). This increase in GlcChol was accompanied by a similar elevation in total Chol levels (Fig. 3I). The efficacy of glucosidase inhibition was verified by GBA and GBA2 ABP labeling (Fig. 3J, K and supplemental Fig. S3E). We cannot exclude that GlcChol levels might also be, at least partially, modulated by differences in serum lipoprotein uptake caused by the treatments. Using Gb3 and LacCer levels as a proxy for lipoprotein uptake, we could observe that GBA inhibition and cyclodextrin treatment may indeed increase lipoprotein uptake (supplemental Fig. S3F, G). This fact should be taken into consideration when interpreting the results.

### GlcChol-exposed Mφ are multinucleated and present an impaired inflammatory response

**Fig. 4:**
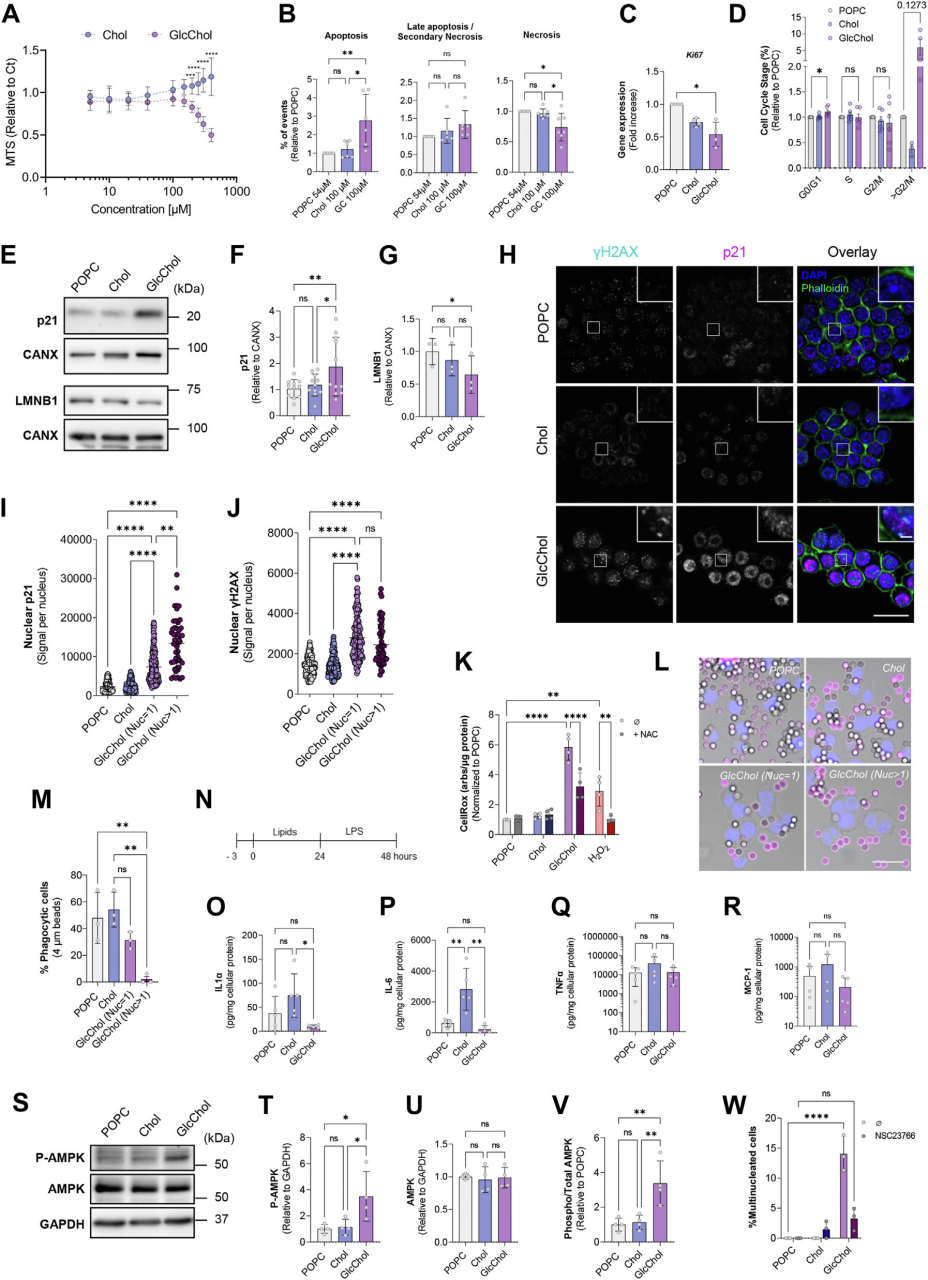
GlcChol impairs the proliferation and inflammatory response of Mϕ. A: Effect of GlcChol:POPC liposomes on RAW 264.7 cell viability. Chol:POPC liposomes were used for comparison. Mϕ were incubated with liposomes for 72 h and cell viability was evaluated with the MTS assay. The results are presented as mean ± SD of 6 independent experiments. The *P* values were obtained by two-way ANOVA-Šidák test; ∗∗*P* < 0.01; ∗∗∗∗*P* < 0.0001. B: Percentage of apoptotic cells (annexin V positive) after 72 h of treatment (Chol:POPC or GlcChol:POPC) normalized to POPC-vehicle control. Results represent the mean ± SD of 6 independent experiments. More than 10k cells were analyzed per experiment. C: mRNA expression levels of *Ki67* in RAW 264.7 cells exposed to POPC (54 μM), Chol (100 μM) and GlcChol (100 μM) for 72 h. mRNA levels were assessed by qRT-PCR. Data were normalized to the endogenous *Gapdh* and *Pgk1* genes. The values are mean ± SD of 4 independent experiments. The *P* values were obtained paired *t* test; ∗*P* < 0.05. D: Flow cytometric analysis of DNA cell cycle after propidium iodide (PI) staining in RAW 264.7 cells after treatment with liposomes for 72 h. Data represents the percentage of cells on a given cell cycle stage. The values are mean ± SD of 4–6 independent experiments. The *P* values were obtained paired *t* test; ∗*P* < 0.05. Immunoblots (E) and densiometric quantification of the protein levels of p21 (CIP1/WAF1) (F) and Lamin B1 (LMNB1) (G) in lysates of cells treated with POPC, Chol or GlcChol for 72 h. Data = mean ± SD of 3–10 independent experiments; ∗*P* < 0.05; ∗∗*P* < 0.01 (two-way ANOVA-Tukey test). H: Representative images of γH2AX and p21 in RAW 264.7 Mϕ incubated with 100 μM GlcChol or with control liposomes for 72 h. Nuclei were labeled with DAPI and F-actin with Phalloidin 488. Confocal single-slice images. The insets are enlargements of the areas outlined with the white boxes. Scale bars, 10 and 2 μm in the insets. Quantification of γH2AX (I) and p21 (J) nuclear signal intensity in RAW 264.7 cells exposed to POPC, Chol and GlcChol for 72 h. The results are mean ± SD of 3 independent experiments. At least, 15 cells were analyzed per experiment. The *P* values were obtained by Kruskal-Wallis with Tukey post test; ∗∗*P* < 0.01; ∗∗∗∗*P* < 0.0001. K: Levels of ROS in RAW cells exposed to the different liposomes assessed by fluorometric assay with the CellRox probe in a 96 well format. H_2_O_2_ and N-acetylcysteine (NAC) were used as positive and negative controls, respectively. Data = mean ± SD of 3–4 independent experiments; ∗*P* < 0.05; ∗∗*P* < 0.01 (Two-way ANOVA-Tukey test). L: Representative images of RAW 264.7 Mϕ exposed to the liposomes (POPC, Chol, or GlcChol) for 72 h and then fed with IgG-opsonized beads for 30 min. Nuclei were stained with DAPI (blue) and non-internalized beads stained with an anti-human IgG antibody (magenta). Confocal single-slice and bright field images are overlayed. Scale bar, 10 μm. M: Quantification of the percentage of cells that present at least one IgG-opsonized particle. The values are means ± SD of 3 independent experiments. 15 cells were analyzed per condition. ANOVA-Tukey test; ∗∗*P* < 0.01. N: Experimental set-up for cytokine assessment. Three hours after seeding the cells were exposed to liposomes (POPC, Chol e GlcChol) for 24 h. The media was then replaced by fresh media with 100 ng/ml LPS for an additional 24 h. Cytokine IL-1α (O), IL-6 (P), TNF-α (Q), and chemokine MCP-1 (R) levels (picogram per milligram of cellular protein) in the media of Mϕ pre-conditioned with liposomes for 24 h and then exposed to LPS (100 ng/ml) for 24 h. Cytokine and chemokine levels were assessed by flow cytometry multiplex assay. Data = mean ± SD of 5 independent experiments; ns = nonsignificant; ∗*P* < 0.05; ∗∗*P* < 0.01; (Two-way ANOVA-Tukey test). Immunoblots (S) and densiometric quantification of the protein levels of phosphorylated-AMPK (Thr172) (T), AMPK (U), phosphorylated/total AMPK ratio (V) in lysates of cells treated with POPC, Chol or GlcChol for 72 h. Data = mean ± SD of 4 independent experiments, ∗*P* < 0.05, ∗∗*P* < 0.01 (ANOVA-Tukey test). (W) Percentage of polyploid cells after a 72 h treatment with liposomes alone or in combination with 50 μM Rac1 inhibitor NSC23766. Data = mean ± SD of 3–10 independent experiments. At least 50 cells were counted per condition. ∗∗∗∗*P* < 0.0001 (Two-way ANOVA-Tukey test).

Given the propensity of Mφ to store GlcChol, next we decided to establish an *in vitro* model of these cells to test the potential pro-atherogenic properties of GlcChol. We chose to expose murine Mφ RAW 264.7 to POPC liposomes loaded with GlcChol (60:40, GlcChol:POPC), and POPC liposomes were used as vehicle control. In addition, Chol-loaded liposomes (60:40, Chol:POPC) were used to compare the impact of the glucosylated versus free sterol on cellular homeostasis. Exposure to GlcChol:POPC for 72 h caused a significant decrease in RAW 264.7 cell viability for concentrations above 150 μM compared to Chol:POPC liposomes (Fig. 4A). The impact of POPC vehicle liposomes on cell viability has been published in a previous study (5).

Based on our measurements and considering a projected tissue density of 1.22 g/ml for non-calcified atherosclerotic plaques (45), we can estimate local GlcChol concentrations to vary between 1 and 12 μM in the CEA analyzed. However, plaques are composed by multiple cell types and given Mφ high phagocytic capacity and tendency to accumulate GlcChol, we can speculate that local concentrations within these cells likely exceed the average tissue values. Therefore, we decided to use a concentration of 100 μM at which no significant differences in cellular metabolic activity (MTS assay) were observed. Despite the inherent limitations of the model, we propose that 100 μM GlcChol over a 3-day period may reasonably reflect the microenvironmental burden experienced by Mφ over the decade-long process of advanced lesion development. Firstly, we evaluated the viability of cells exposed to 100 μM of GlcChol by performing the Annexin-V-PI staining for flow cytometry. Exposure of Mφ to GlcChol caused a significant increase in cells undergoing apoptosis in parallel with a decrease in necrotic cells, when compared to control cells exposed to POPC (vehicle) or Chol:POPC (Fig. 4B). Additionally, we observed that GlcChol-exposed cells presented decreased *Ki67* mRNA levels, a proliferative cell marker, compared to POPC and Chol-treated cells (Fig. 4C). This suggested a decrease in cell proliferation, which prompted us to analyze the cell cycle of Mφ exposed to the liposomes by flow cytometry with propidium iodide (PI). In cells exposed to GlcChol, we observed a small but significant increase in the fraction of cells at the G0/G1-phase, when compared to the two control conditions (Fig. 4D and supplemental Fig. S4A, B). Interestingly, we detected a considerable (albeit not significant due to the dispersion of the values) increase in the fraction of cells at > G2/M, which in this assay corresponds to the presence of polyploid cells (Fig. 4D). In certain pathologic conditions, such as foreign body reactions and peripheral inflammatory lesions, monocytes are known to fuse to form large, multinucleated giant cells (46).

Analysis of the protein levels of cyclin-dependent kinases (CDK) inhibitors, which control RNA transcription and cell cycle progression, revealed a significant increase in tumor suppressor p21/CDKN1A (Fig. 4E, F) but not in p16 (not shown). Expression of p21 can be upregulated by viral infection or induction of DNA damage, following the activation of the p53 transcription factor (47). At the time point analyzed (72 h), we did not detect alterations in total p53 protein levels (data not shown). Of note, p21 is known to negatively regulate the G1/S transition (48), which could explain the minor G0/G1 arrest observed before (Fig. 4D). While cytoplasmic p21 activity favors cell growth and survival, its nuclear concentration promotes cell cycle arrest and inhibition of growth (47). Accordingly, we observed a striking nuclear accumulation of p21 protein in cells exposed to GlcChol compared to control cells (Fig. 4H). Furthermore, the confocal images confirmed the existence of a significant number (circa 15%) of multinucleated cells in the GlcChol-treated condition. Interestingly, the p21 nuclear buildup was even more pronounced in polyploid cells compared to monoploid GlcChol-treated cells (Fig. 4H, I). One of the major triggers of p21 nuclear activity is DNA damage caused by genotoxic insults such as oxidative stress and UV radiation (47).

To investigate whether DNA damage was occurring in Mφ exposed to GlcChol, we assessed the protein levels of nuclear membrane protein Lamin B1 and phosphorylated (Ser-139) γH2AX histone. Lamin B1 (LMNB1) is a cellular senescence marker whose protein levels are known to decrease in response to DNA damage (49). Exposure to GlcChol caused a 50% decrease in LMNB1 protein levels in comparison to POPC control (Fig. 4E, G). γH2AX is a sensitive marker for DNA damage which correlates with double-strand breaks (50). Accordingly, we also detected a significant increase in the presence of γH2AX foci in mono- and multinucleated GlcChol-treated cells compared to controls (Fig. 4H, J), suggesting the occurrence of DNA damage upon the exposure to the glucosylated sterol. To assess whether this damage was being caused by oxidative stress mediated by reactive oxygen species (ROS), we evaluated general ROS levels with the CellRox probe, which detects superoxide and hydroxyl radicals. Exposure to GlcChol for 72 h caused a 6-fold increase in detected ROS levels in Mφ compared to both controls (Fig. 4K). These levels were comparable to those triggered by the positive control hydrogen peroxide (H_2_O_2_) and were fully preventable by incubation with the antioxidant ROS-scavenger N-acetylcysteine (NAC) (Fig. 4K). Additionally, mRNA levels of the oxidative stress regulator Heme oxygenase-1 (*Hmox1*) were also significantly elevated in GlcChol-treated cells compared to controls (supplemental Fig. S4D). Thus, we show a direct connection between exposure of Mφ to GlcChol, ROS-mediated DNA damage, p21 nuclear translocation, and impaired cellular proliferation.

Besides its role in cell cycle arrest, p21 also plays a central role in the regulation of innate and adaptive immunity. P21 negatively regulates the Mφ inflammatory response by mediating Mφ reprogramming (51). Taking this into account, together with the fact that multinucleated cells are known to be less responsive to inflammatory stimulation (52), we decided to investigate the inflammatory response of Mφ exposed to GlcChol. First, we evaluated FcγR receptor-mediated phagocytosis of IgG-coated particles as a readout for functional phagocytic capacity and immune cell activation. Approximately 40% of POPC and Chol-treated Mφ were able to phagocytise IgG-coated particles following a 30 min pulse (Fig. 4L, M), this number was approximately 30% in mononucleated GlcChol-exposed Mφ (this difference was not significant). In contrast, the phagocytic index of multinucleated GlcChol-treated cells was close to zero (*P* < 0.01 vs. POPC and Chol controls), suggesting an impairment of the immune response of these cells.

We then evaluated Mφ cytokine production upon exposure to the different liposomes. At 72 h incubation with the 3 types of liposomes, there were no significant differences in the cytokines TNF-α, and IL-10 released into the cell culture media, although the transcript levels of these cytokines were increased in GlcChol-exposed cells (supplemental Fig. S4H–K). We also observed a significant decrease in the expression of several genes (*Cd11b*, *Cd74* and *Klf4*) associated with Mφ polarization towards a pro-inflammatory phenotype (53, 54, 55) (M1, classical activation) (supplemental Fig. S4E–G). Additionally, when RAW 264.7 cells were pre-conditioned with liposomes for 24 h and then stimulated with the pro-inflammatory agent Lipopolysaccharide (LPS) (Fig. 4N), we could observe a significant decrease in IL-6 and IL-1α cytokine production in GlcChol-compared to Chol-exposed cells (Fig. 4O, P). While Chol treatment primed the immune cells to produce these pro-inflammatory cytokines, GlcChol failed to do so, with cytokine levels even falling behind vehicle (POPC) levels, albeit not significantly. The levels of the cytokine TNF-α and the chemokine MCP-1 (Monocyte chemoattractant protein-1) were similar between these 3 conditions (Fig. 4Q, R).

Next, we investigated the activity of several signaling pathways involved in the modulation of Mφ immune responses, in the hope of elucidating the pathway underlying the GlcChol-mediated inhibition of immune responses. Analysis of the transcriptional activator NF-κB (nuclear factor κB) signaling pathway revealed significant decreases in Toll-like receptor 4 (TLR4) and TNF-α cellular protein levels in GlcChol-treated cells compared to controls (supplemental Fig. S4L, M, T), in line with previous observations suggesting an impaired immune response. Nonetheless, the analysis of the activation state of NF-κB and its downstream effector IκBα was inconclusive (supplemental Fig. S4L, N–S). The STAT3 (Signal Transducer and Activator of Transcription 3) signaling pathway also showed no clear signs of modulation by exposure to GlcChol at the 72 h time point (supplemental Fig. S4L, U–X). In contrast, we observed a striking activation of the AMP-activated protein kinase (AMPK) signaling pathway in Mφ exposed to GlcChol for 72 h, namely a 3-fold increase in the ratio of phosphorylated to total AMPK protein compared to controls (Fig. 4S–V). Of note, this kinase is known to regulate cell cycle through the up-regulation of the p53-p21 axis (56), in agreement with our previous observations.

Nonetheless, the decrease in proliferation and cell cycle arrest triggered by GlcChol in these cells are relatively modest and may not fully attest for the occurrence of the multinucleated cells upon GlcChol treatment. Therefore, we set out to investigate whether these cells might be formed through fusion events. In certain pathologic conditions, such as foreign body reactions and peripheral inflammatory lesions, monocytes are known to fuse and form large, multinucleated cells (57). Previous studies have reported the fusion of Mφ during foreign body response and osteoclast differentiation to be dependent on the activity of the Rho GTPase Ras-related C3 botulinum toxin substrate 1 (Rac1) (58, 59, 60, 61). Addition of the Rac1 inhibitor NSC23766 to the culture media in simultaneous with the liposome treatment caused a significant decrease in the percentage of multinucleated cells upon exposure to GlcChol, from circa 15% of polyploid cells to less than 5% (Fig. 4W). Thus, RAW 264.7 Mφ fusion in response to GlcChol seems, at least partially, Rac1 dependent.

### Exposure to GlcChol impacts the late endosome/lysosome compartment of Mφ

**Fig. 5:**
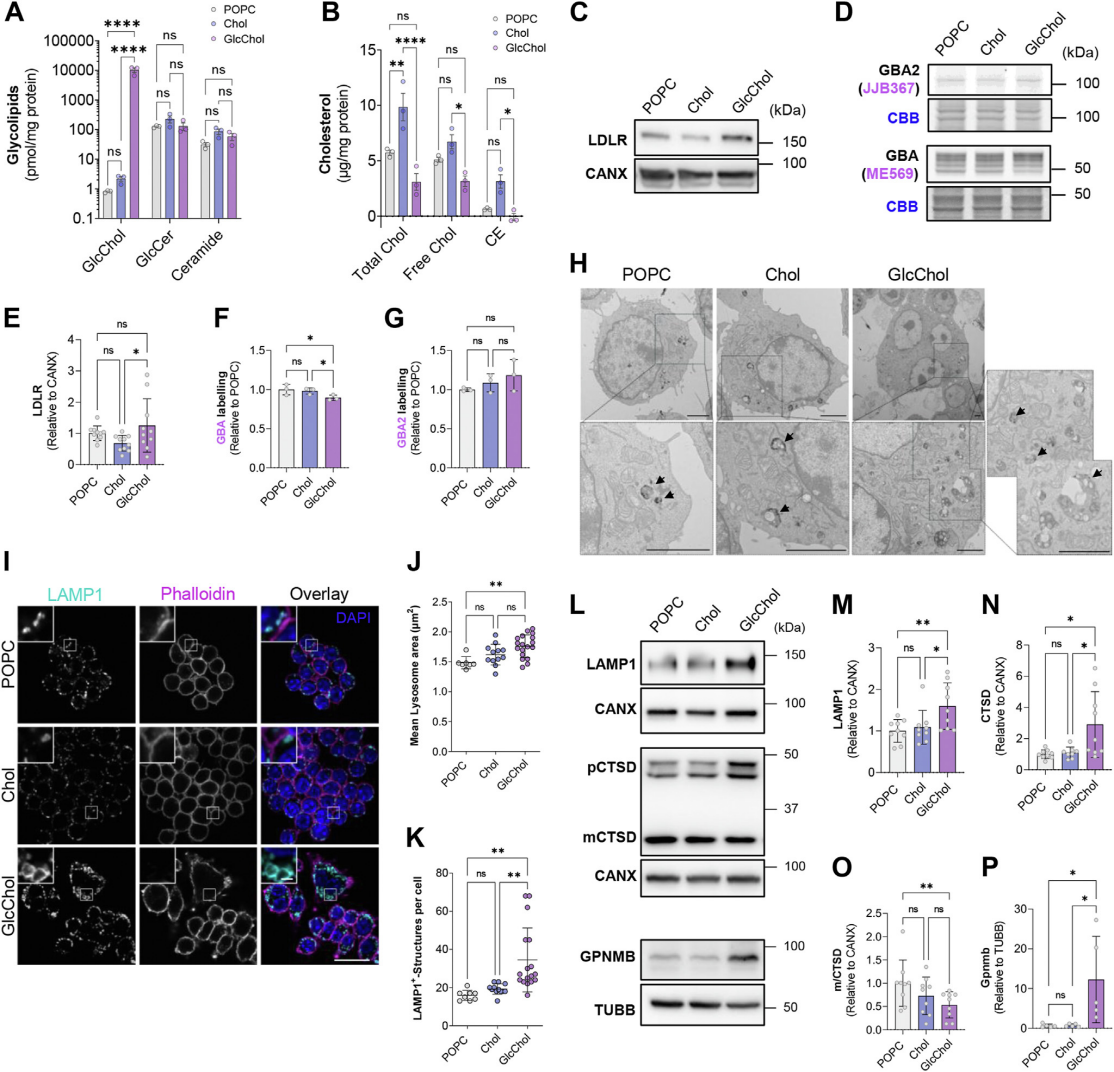
GlcChol impacts the late endosome/lysosome compartment. A: Levels of GlcChol, glucosylceramide and ceramide determined by LC-MS/MS (picomole per milligram of protein) in lysates of RAW 264.7 cells exposed POPC, Chol or GlcChol for 72 h. The results are mean ± SD of 3 independent experiments. B: Fluorometric quantification of total Chol, unesterified (free) Chol levels and esterified cholesterol (microgram per milligram of protein) in lysates of Mϕ exposed for 72 h to 54 μM POPC (vehicle), 100 μM Chol or 100 μM GlcChol. The results are mean ± SD of 3 independent experiments. The *P* values were obtained by two-way ANOVA-Tukey test; ∗*P* < 0.05; ∗∗*P* < 0.01; ∗∗∗∗*P* < 0.0001. Immunoblot (C) and densiometric quantification (E) of LDLR protein levels in lysates of cells treated with POPC, Chol or GlcChol for 72 h. Protein levels were normalized to calnexin (CANX) levels. Data = mean ± SD of 10 independent experiments, ∗*P* < 0.05 (ANOVA-Tukey test). Fluorescent labeling (D) and quantification of active GBA with the ABP ME569 (F) and GBA2 with the ABP JJB367 (G) in lysates of cells exposed to POPC, Chol or GlcChol for 72 h. Gels were stained with Coomassie Brilliant Blue (CBB) as loading control. Data = mean ± SD of 3 independent experiments, ∗*P* < 0.05 (ANOVA-Tukey test). H: Transmission electron microscopy of RAW 264.7 cells treated for 72 h with POPC, Chol or GlcChol liposomes. GlcChol-treated cells exhibit larger lysosomes as confirmed by BSA-gold labeling (bottom row). Dark arrows indicate gold-containing endolysosomes. Scale bars: 2 μm in all images. I: Representative images of LAMP1 in RAW 264.7 Mϕ incubated with 100 μM GlcChol, POPC or Chol liposomes for 72 h. Nuclei were labeled with DAPI and F-actin with Phalloidin 633. Confocal single-slice images. The insets are enlargements of the areas outlined with the white boxes. Scale bars: 10 and 2 μm in the insets. Quantification of mean lysosome area (J) and lysosome number of lysosomes (K) (LAMP-1 positive structures) in Mϕ pulsed for 72 h with POPC, Chol or GlcChol. Quantification was performed using a CellProfiler script. The results are mean ± SD of 3 independent experiments. At least 3 fields cells were analyzed per experiment (>10 cells per field). The *P* values were obtained by ANOVA-Tukey test. ∗∗*P* < 0.01. Immunoblots (L) and densiometric quantification of the protein levels of LAMP1 (M), CTSD (N), mature/total CTSD ratio (O) and gpNMB (P) in lysates of cells treated with POPC, Chol or GlcChol for 72 h. Protein levels were normalized to calnexin (CANX) or tubulin (TUBB) levels. Data = mean ± SD of 5–9 independent experiments, ∗*P* < 0.05; ∗∗*P* < 0.01 (Two-way ANOVA-Tukey test).

To gain a better understanding of the molecular mechanisms underlying this phenotype, we decided to zoom in on the alterations caused by GlcChol exposure in Mφ at the level of lipid and organelle homeostasis. As expected, GlcChol levels were significantly increased in the cells exposed to this sterol in comparison to control cells, thereby confirming the uptake of the liposomes (Fig. 5A). However, we could not determine the exact intracellular location of the lipid. Levels of the sphingolipids ceramide and glucosylceramide (GlcCer) were similar between the three conditions analyzed (Fig. 5A). Curiously, exposure to GlcChol was unable to raise total cholesterol levels as observed in cells exposed to Chol:POPC liposomes (Fig. 5B), suggesting that free cholesterol is not being released by hydrolysis following the cellular uptake of GlcChol or that it is being effluxed by the ABCA1 transporter. This is mirrored in the protein levels of LDL receptor (LDLR), which decrease in response to Chol:POPC but fail to do so in reaction to GlcChol (Fig. 5C, E). This is in line with the expression levels of other genes involved in cholesterol homeostasis (*Abca1*, *Lipa* & *Hmgr*) that are also regulated by intracellular Chol levels (supplemental Fig. S5A–C). Next, we evaluated the levels of active glucosidases GBA and GBA2 in these cells by using specific activity-based probes directed against each of these enzymes. While GBA2 activity levels were unaltered by exposure to GlcChol (Fig. 5D, G), active GBA levels were slightly but significantly reduced (Fig. 5D, G), suggesting an impact on the lysosomal compartment. GBA2 (but not GBA) transcript levels were also significantly reduced in GlcChol-treated compared to POPC-treated cells, which may represent a response mechanism to the excess of cellular GlcChol in these Mφ (supplemental Fig. S5E, F).

To evaluate the impact of the GlcChol liposomes on late endosomes/lysosomes (LE/L) we labelled this cellular compartment by means of a pulse-chase experiment with BSA conjugated with gold particles. This conjugate is taken up by endocytosis and follows the degradative pathway, allowing the visualization of gold-labeled LE/L upon electron microscopy (EM) analysis. Confirming our previous results, we were able to observe that GlcChol-treated Mφ were often enlarged and presented multiple nuclei. EM-images clearly show that these polyploid cells are around 3 to 4 times larger than Chol- and POPC-treated control cells (Fig. 5H). Gold-labeled LE/L compartments (indicated by the dark arrows) were also enlarged in cells exposed to GlcChol compared to both controls. These enlarged lysosomes in GlcChol-treated RAW 264.7 Mφ also presented signs of storage (Fig. 5H).

Accordingly, lysosomes immuno-labeled with anti-LAMP-1 antibodies and analyzed by confocal microscopy, presented an enlarged morphology compared to controls (Fig. 5I), albeit being devoid of neutral lipids (assessed by BODIPY staining, data not shown). Quantification of these images revealed a significant increase in LE/L mean area when compared to vehicle control (Fig. 5J) and in the number of organelles (LAMP1-positive structures) compared to both POPC and Chol controls (Fig. 5K). Additionally, we detected several indications of lysosomal stress in cells exposed to 100 μM of GlcChol, namely an increase in lysosomal-associated protein 1 (LAMP-1) (Fig. 5L, M) and gpNMB (Fig. 5L, P) protein levels, as well as a significant decrease in the mature to total cathepsin-D (CTSD) levels (Fig. 5L, N, O), suggesting impaired lysosomal proteolysis (3, 5). Analysis of other organelles, namely mitochondria, peroxisomes, Golgi apparatus, early endosomes and lipid droplets, by immunoblot and immunofluorescence did not reveal any significant alterations apart from a significant decrease in the protein levels of the Succinate Dehydrogenase (SDHB), also known as the Iron-Sulfur Subunit of Complex II of the mitochondrial oxidative phosphorylation pathway (supplemental Fig. S5G–N).

### Exposure to GlcChol causes mTORC1 activation in parallel with TFEB translocation

**Fig. 6:**
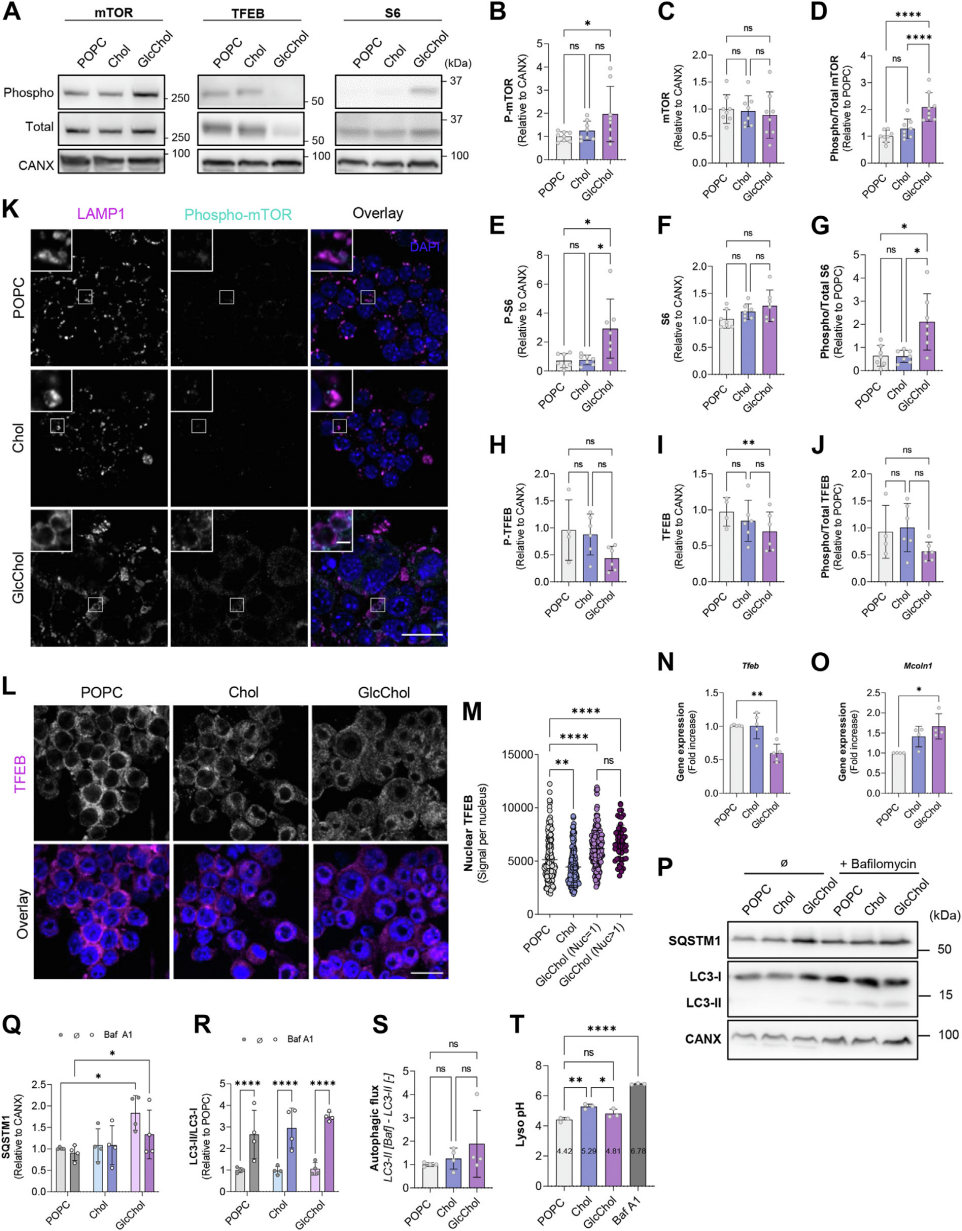
Exposure to GlcChol activates the mTOR signaling pathway in murine Mϕ. Immunoblots (A) and densiometric quantification of the protein levels of phosphorylated-mTOR (Ser2448) (B), mTOR (C), phosphorylated/total mTOR ratio (D), phospho-S6 Ribosomal Protein (Ser235/236) (E), S6 (F), phosphorylated/total S6 ratio (G), phosphorylated TFEB (Ser211) (H), TFEB (I), phosphorylated/total TFEB ratio (J), in lysates of cells treated with POPC, Chol or GlcChol liposomes for 72 h. Protein levels were normalized to calnexin (CANX) or GAPDH levels. Data = mean ± SD of 6–8 independent experiments, ∗*P* < 0.05, ∗∗*P* < 0.01, ∗∗∗∗*P* < 0.0001 (ANOVA-Tukey test). K: representative images of LAMP1 and phosphorylated-mTOR (Ser2448) in RAW 264.7 Mϕ incubated with 100 μM GlcChol or with control (POPC or Chol) liposomes for 72 h. (L) Representative images of TFEB in GlcChol-treated cells and controls. Nuclei were labeled with DAPI. Confocal single-slice images. The insets are enlargements of the areas outlined with the white boxes. Scale bars, 10 and 2 μm in the insets. (M) Quantification of TFEB nuclear signal intensity in RAW 264.7 (72 h treatment). The results are mean ± SD of 3 independent experiments. At least, 15 cells were analyzed per experiment. The *P* values were obtained by one-way Kruskal-Wallis with Tukey post-test; ∗∗*P* < 0.01; ∗∗∗∗*P* < 0.0001. mRNA expression levels of *Tfeb* (N) and *Mcoln1* (O) genes. mRNA levels were assessed by qRT-PCR. Data were normalized to the endogenous *Gapdh* and *Pgk1* genes. The values are mean ± SD of 5 independent experiments. The *P* values were obtained by paired *t* test; ∗*P* < 0.05; ∗∗*P* < 0.01. P: immunoblot of LC3B and p62/SQSTM1 in cell lysates of control and GlcChol-treated cells for 72 h, without or with Bafilomycin A1 (Baf A1) treatment (2 h). Q: Quantification of the p62/SQSTM1 levels (normalized to CANX) in cell lysates of POPC, Chol and GlcChol-treated cells at 72 h. R: Quantification of the LC3-II/LC3-I ratio at 72 h (relative to POPC). S: Autophagic flux (calculated as the difference between LC3-II levels in the presence and absence of Baf A1) in RAW cells treated with GlcChol and 72 h (relative to POPC). Data = mean ± SD of 4 independent experiments, ∗∗*P* < 0.01; ∗*P* < 0.05 (ANOVA-Tukey test). T: Quantification of lysosomal pH in live cells measured by ratiometric fluorescence. Baf A1 was used as positive control. Three independent experiments were performed and each time 20 lysosomes were analyzed. Error bars represent SD of 3 independent experiments. ∗*P* < 0.05, ∗∗*P* < 0.01 (two-way ANOVA-Tukey test).

The impact of GlcChol on the lysosome is reflected in the activation of the mammalian target of rapamycin (mTOR) signaling pathway. After 72 h of exposure to GlcChol we observed a significant increase in phosphorylated mTOR levels compared to POPC or Chol-treated Mφ (Fig. 6A–D). Phosphorylated mTOR was also observed to decorate the membrane of LE/L of cells supplemented with GlcChol, indicating its recruitment to these organelles (Fig. 6K). The levels of phosphorylated S6 protein, a downstream effector of mTORC1, were also significantly elevated in GlcChol-treated cells compared to controls (Fig. 6A, E–G). On the contrary, transcription factor EB (TFEB) phosphorylation levels were reduced, albeit just short of significance, in GlcChol-treated cells as well as total TFEB protein (Fig. 6A, H–J) and transcript levels (Fig. 6N). TFEB is also a downstream effector of mTORC1 and master regulator of lysosomal biogenesis. Immunofluorescence analysis confirmed that de-phosphorylated TFEB significantly translocated to the nucleus in Mφ exposed to GlcChol compared to POPC and Chol controls (Fig. 5L, M), where it could stimulate the transcription of lysosome and autophagy-associated genes. Even at this relatively late time point, we could still observe a significant increase in the transcript levels of the CLEAR-element containing gene (62) *Mcoln1* (Fig. 6O). To further investigate the downstream consequences of mTORC1 activation and TFEB translocation, we evaluated the autophagic flux under these conditions by treating the lipid-exposed cells with Bafilomycin A1, a potent inhibitor of the lysosomal v-ATPase (responsible for maintaining an acidic intraluminal pH). We noted that SQSTM1 protein levels were significantly elevated in cells exposed to GlcChol compared to controls (Fig. 6P, Q). However, despite minor alterations the microtubule-associated proteins 1A/1B light chain 3B (LC3)-II to LC3-I ratio and the overall autophagic flux, there were no significant changes in these parameters when compared to control levels (POPC and Chol) (Fig. 6P, R, S). Accordingly, assessment of the lysosomal pH with a ratiometric method based on the uptake of fluorophore-labeled dextran did not reveal a significant alteration in GlcChol-exposed cells compared to POPC controls for this parameter (Fig. 6T). As previously described (63), exposure to exogenous Chol significantly alkalinized the intralysosomal pH. In agreement with these results, we also did not find any signs of damage to the lysosomal membrane (Galectin-3 puncta) at the time point studied (data not shown). Altogether, despite directly impacting the LE/L compartment and inducing some signs of lysosomal stress, GlcChol does not seem to fully inhibit lysosomal catabolic activity nor autophagy.

Of note, to exclude the possibility that this phenotype was exclusively elicited in RAW 264.7 cells, we have validated our most important observations in mouse J774.1 macrophage-like cells. Upon exposure to 100 μM of GlcChol these cells presented a similar phenotype to RAW cells, namely: presence of giant multinucleated cells, absence of neutral lipid accumulation, lysosomal mass increase, impaired CTSD maturation and p21 upregulation, and lack of cytokine production (supplemental Fig. S6).

In summary, the GlcChol accumulated at the core of atherosclerotic lesions has the potential to strongly impact Mφ homeostasis and even lead to cell death at high concentrations. Under this threshold, cells appear to activate two parallel protective mechanisms. On the one hand, activation of the lysosome-AMPK-p21 axis seems to lead to decreased cell proliferation and partial cell cycle arrest, thereby countering apoptosis. On the other hand, the Rac1-mediated fusion of Mφ may hinder their immune activation, preventing an aggravation of pro-inflammatory responses.

## Discussion

Our study brings further evidence that atherosclerosis may be seen as a local acquired lysosomal storage disorder (LSD). Much like the tissues of LSD patients, the core of atherosclerotic lesions accumulates storage material as a consequence of the exhaustion of the degradative capacities of the lysosomes (64). In particular, we confirmed previous evidence showing that lesions accumulate cholesterol (Chol) and glycosphingolipids (GSLs), storage materials of LSDs such as Gaucher disease and Niemann-Pick type A and type C (NPA and NPC). The pathology of these inherited disorders is, like atherosclerosis, partially mediated by the formation of lipid-laden foam cells, characterized by a lysosomal accumulation of undegraded lipids in the late endosomal/lysosomal (LE/L) compartment. We thus set out to investigate whether atherosclerotic foam cells could form the same type of secondary metabolites observed in the LSD context, as a response to the primary storage accumulation. We provide evidence that atherosclerotic lesions indeed accumulate lyso-glycosphingolipids, such as glucosylsphingosine and lactosylsphingosine, which are formed by the action of the lysosomal hydrolase acid ceramidase upon lysosomal storage of the respective GSL counterparts, glucosylceramide (GlcCer), and lactosylceramide (LacCer). The difference being that, unlike what occurs in LSDs, this accumulation appears to be exclusively local and not systemic, seeing as the levels of these lyso-glycosphingolipids remain unaltered in the circulation of patients who suffer from CVDs related to atherosclerosis.

Our previous work brought to light the fact that in Gaucher and NPC foam cells, the lysosomal storage of Chol and GlcCer stimulates the production of glucosylated cholesterol (GlcChol) via a transglucosylation reaction catalyzed by the glucocerebrosidase (GBA) enzyme (33). The occurrence of this lipid has so far not drawn significant attention, with only a couple of studies existing that allude to its potential function in the mammalian context (24, 28, 65, 66, 67). We demonstrate that like its precursors, GlcCer and Chol, GlcChol also accumulates in atherosclerotic plaques. However, like lyso-GSLs this local accumulation is not reflected in an elevation of lipoprotein-bound GlcChol in the circulation of CVD patients suffering from atherosclerosis. Nonetheless, the elevation in the GlcChol to total Chol ratio in the plasma of myocardial infarction patients (without previous prescription of statins), may suggest that an elevated local GlcChol synthesis may have a systemic reflection. Thus, more studies are needed to ascertain whether the GlcChol/Chol ratio could be used as a proxy for lesion development and local lipid storage in atherosclerosis.

Considering that plaque foam cells may be derived from lipidotic macrophages (Mφ) or vascular smooth muscle cells (VSMCs), we then set out to investigate whether both types of cells were able to produce GlcChol in atherosclerosis *in vitro* models. For that, we employed a single component of oxLDL, cholesteryl hemiazelate (ChA), previously shown by us to trigger foam cell formation in both cell types (3, 5). VSMC-derived foam cells showed a two-fold increase in Chol levels and a decrease in GlcCer upon ChA-triggered foam cell conversion. Consequently, GlcChol levels were not elevated in this model. In sharp contrast, the 3-fold Chol build-up in Mφ -derived foam cells was sufficient, even in the absence of GlcCer storage, to cause a significant rise in GlcChol levels. These differences between foam cells from different origins could be ascribed to the higher degree of fluid-phase and receptor-mediated endocytosis in Mφ compared to VSMCs, as well as to a higher lysosomal hydrolytic capacity in the former. In fact, VSMCs are known to possess much lower levels of lysosomal lipase (LAL) required for the processing of cholesteryl esters (CE) and hemiesters (ChE) within the lumen of the lysosomes (68, 69). So, the lower levels of free Chol and, consequently, GlcChol in VSMCs-derived foam cells may be due to a lower rate of CE and ChE hydrolysis. Moreover, in our Mφ foam cell model, the accumulation of GlcChol appears to derive from the partial inactivation of GBA caused by exposure to ChA, which hinders the lysosomal degradation of the glycolipid. Accordingly, the build-up of GlcChol in ChA-treated cells was further aggravated by inhibition of GBA with CBE. This leads us to suggest that GlcChol might be accumulating in the lysosomes of Mφ foam cells due to its impaired hydrolysis. The concomitant inhibition of GBA2 triggered by exposure to ChA may represent a feedback mechanism to reduce GlcChol synthesis, as previously observed in the case of AMPK-mediated GCS inhibition (70). In the future, it would be interesting to compare the effect of ChA on GlcChol metabolism with that of more complex lipoprotein formulations, namely oxLDL. Having established that Mφ are the plaque cell type most likely to drive GlcChol accumulation, we then set out to investigate its role in atherogenesis. Studies addressing the physiological role of GlcChol in mammalian systems are scarce and point in different directions. Links have been established with heat-shock response (66), *Helicobacter pylori* infection (71), neuronal and glial extracellular vesicle formation (22), neuronal apoptosis (28, 72) and oxidative stress (65). Given the complexity of GlcChol metabolism, which is dependent on local availability of substrates to two different enzymes, we decided to employ a simpler system to study the impact of GlcChol in Mφ homeostasis: delivery via GlcChol:POPC liposomes. Our studies showed that GlcChol concentrations above 150 μM significantly decrease RAW 264.7 cell viability. Therefore, we decided to employ a concentration of 100 μM to analyze the impact of this lipid. GlcChol induces some degree of apoptosis at this concentration as well as a decrease in cell proliferation, but no significant difference in metabolic activity assessed by MTS assay.

One of the striking phenotypes observed in Mφ exposed to GlcChol was the occurrence of a significant number of enlarged polyploid cells. The origin of these cells seems to be a Rac1-mediated fusion of the Mφ, as previously reported (58). Mφ often undergoes fusogenic processes, constituting the primary source of the giant multinuclear cells (GMC) observed in aging (73). The occurrence of GMCs is accounted for in the context of cancer, foreign-body recognition, osteoclast formation, Langhans giant cells, and, interestingly, giant-cell arteritis (46, 58, 73, 74, 75, 76). A previous study has demonstrated that GMCs can be formed by IL-4 stimulation and that these cells, despite preserving Mφ surface protein expression, lose the ability to mount a cytokine response upon LPS stimulation (52). This is in complete agreement with our observations that GlcChol-derived GMCs are particularly impaired in producing an immune response, such as FcγR receptor-mediated phagocytosis and cytokine production upon LPS stimulation.

We propose a model in which GlcChol affects Mφ homeostasis by triggering ROS formation, possibly due to a decrease in the complex II of the mitochondrial electron transport chain. Additional hypothetical ROS sources are the lysosomal NADPH oxidase, iron-mediated Fenton reactions triggered by impaired lysosomal iron metabolism or altered lysosome-mitochondria interactions (i.e., metabolite exchange via contact sites). The increase in ROS causes DNA damage, which in turn causes the recruitment of p21 to the nucleus, where it can mediate DNA repair (77). In Mφ, p21 regulates the cell cycle and prevents apoptosis, acting as a negative regulator of Mφ activation (78). As previously described (46, 77, 79), p21-mediated cell cycle arrest and Rac1-mediated GMC formation may represent parallel and interconnected mechanisms by which Mφ react to the cellular stress elicited by exposure to GlcChol. In cellular stress conditions, p21 is known to act as an early signal, priming cells for immunosurveillance by recruiting Mφ to stressed cells as part of an immediate early response (80). This study directly linked p21’s role in cell cycle arrest with its ability to mediate immune responses. The regulation of p21 expression can occur through pathways independent of p53, which could explain why we did not see an upregulation of this protein (at the time point analyzed). For instance, p21 is induced via a p53-independent pathway during ceramide-mediated G1 arrest in hepatocarcinoma cells (81). Our observations also suggest that p21 upregulation and G0/G1 cell cycle arrest could be caused by signaling via AMPK, as observed previously in human aortic smooth muscle cells and rabbit aortic strips (56, 82). Interestingly, cells are known to suppress GlcCer synthesis via AMPK activation (70). Given that GlcCer is needed for GlcChol formation, exposure to GlcChol may also drive AMPK activation as a compensatory mechanism to cope with intracellular GlcChol build-up. Together with mTOR, AMPK plays a major role in governing cellular metabolic programs. Both signaling complexes can be localized at the LE/L membrane surface and are dependent on the v-ATPase-Ragulator for activation (83). However, AMPK can be activated by adenosine monophosphate (AMP)-dependent and -independent mechanisms. The different compartmentalized pools of AMPK are also differentially activated depending on the severity of nutrient or energy stress (84).

We further show that GlcChol directly impacts the LE/L compartment and sterol homeostasis. Cells exposed to GlcChol appear deprived of Chol, as demonstrated by the elevated protein levels of LDLR and mRNA transcripts of *Hmgr*, encoding for one of the rate-limiting enzymes in Chol synthesis (HMG-CoA reductase). Accordingly, the expression levels of the Chol exporter *Abca1* were also reduced. Given that GlcChol strongly impacted LE/L homeostasis it is possible that the cells are unable to hydrolyze the exogenous GlcChol into free Chol and glucose due to an impairment of the lysosomal degradative capacity. Such an impairment in hydrolase activity is corroborated by the decrease in mature (active) cathepsin-D protease and GBA activity, as well as by the occurrence of intralysosomal storage material observed by EM. Nonetheless, lysosomal pH was not significantly impacted, and neither was there any evidence of lysosomal neutral lipid accumulation. Although GlcChol levels increased following exposure to the liposomes, we cannot presently ascertain the intracellular location of the sterol, which may not be reaching the LE/L for effective degradation.

It is clear, however, that some degree of lysosomal storage occurs upon exposure to GlcChol, since we observed an upregulation of lysosomal stress marker gpNMB, also used as a pathological marker for several LSDs (43, 44), and an activation of the mTOR signaling pathway. Judging by the recruitment of phosphorylated mTOR to the lysosomal membrane and the activation of its downstream effector S6 kinase, an activation of the mTOR complex 1 (mTORC1) is probable. However, as reported by Napolitano *et al.* (85), mTORC1 can differently modulate the activity of its downstream effectors differently, which explains why we observe a parallel dephosphorylation of TFEB and its nuclear translocation. GlcChol-treated Mφ appear to react to the lysosomal stress and impaired degradative capacity by upregulating the transcription of lysosomal genes. This, in turn results in the expansion of the lysosomal compartment, observed in the number and mean area of lysosomes, upon GlcChol treatment. Autophagy, on the other hand, does not appear to be stimulated by TFEB translocation, even though there is a build-up of p62/SQSTM1-labeled proteins. The hyperactivation of mTOR may be countering this effect by directing cells towards protein synthesis and autophagy inhibition via S6K signaling (86). In this regard, mTOR and AMPK are known to have opposing effects by stimulating, respectively, cellular anabolism and catabolism, via processes modulated by the lysosomal V-ATPase-Ragulator complex. However, some reports suggest that under conditions of some energy stress (activating AMPK) and nutrient availability (activating mTOR), both pathways may be concomitantly modulated to sustain autophagy during cell growth (87). The degree of cellular stress may also activate different compartmentalized pools of AMPK (84). In the future, it would be of interest to understand the molecular alterations triggered by GlcChol at the organelle level, which may elicit these effects, i.e., DNA damage, mTOR, and AMPK activation.

In conclusion, under a certain threshold, Mφ seems to be able to cope with GlcChol-induced cellular stress by triggering a lysosome-AMPK-p21-dependent signaling pathway that protects cells from apoptosis, either by cell cycle arrest or direct fusion, hindering pernicious pro-inflammatory responses by these cells. Thus, whether GlcChol exerts pro-atherogenic or protective effects within atherosclerotic lesions will hinge on the determination of local lipid concentrations by future studies. The modulation of p21 activity by GlcChol in Mφ could place this lipid at the center of stress signal integration and immune surveillance orchestration. Validating GlcChol as a regulator of Mφ function could establish it as a promising therapeutic target for atherosclerosis and other inflammatory and stress-related conditions, including LSDs.

## Data availability

All raw data pertaining to the manuscript will be shared upon reasonable request to André R. A. Marques (iNOVA4Health, NOVA Medical School, andre.marques@nms.unl.pt).

## Supplemental data

This article contains supplemental data (88, 89, 90, 91).

## Conflict of interests

The authors declare that they have no conflicts of interest with the contents of this article.
