# Supplementary material for: Glucosylated cholesterol accumulates in atherosclerotic lesions and impacts macrophage immune response
Source: J Lipid Res. 2025 May 15;66(6):100825. doi: 10.1016/j.jlr.2025.100825 (PMC12197965; doi:10.1016/j.jlr.2025.100825)

**Supplementary information**

**Supplementary methods**

Synthesis of 1-*O*-cholesteryl-β-d-glucopyranoside (cholesteryl-β-d-glucoside)


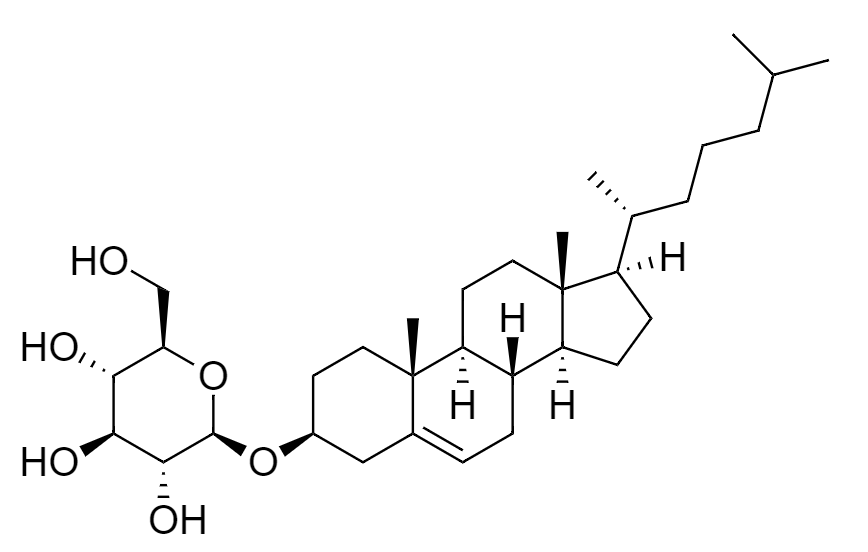


*Chemical Structure 1*: 1-*O*-cholesteryl-β-D-glucopyranoside (cholesteryl-β-D-glucoside)

*Experimental Procedure:* 1-*O*-cholesteryl-β-d-glucopyranoside (cholesteryl-β-d-glucoside) was synthesized from cholesterol and 2,3,4,6-tetra-*O*-acetyl-α-d-glucopyranosyl bromide (acetobromo-α-d-glucose) by adapting previously reported procedures from the literature (19, 87, 88). Zinc oxide (0.88 g; 10.86 mmol) was added to a suspension of cholesterol (2.10 g; 5.43 mmol) and 4 Å molecular sieves (1.20 g) in dry toluene (100 mL). The reaction mixture was refluxed under nitrogen for 24 h to produce the zinc salt of cholesterol. After completion of the reaction, the mixture was cooled to room temperature and a solution of acetobromo-α-d-glucose (1.15 mg; 2.72 mmol) in toluene (40 mL) was added over 30 min. The reaction mixture was refluxed for 24 h and then cooled to RT. After completion of the reaction, the mixture was filtered through a pad of Celite, the solvent was evaporated, and dichloromethane was added. The organic phase was washed with water and saturated aqueous NaCl solution, dried (Na_2_SO_4_) and the solvent evaporated off. The crude product was purified by column chromatography [chloroform/methanol (30:1)] to give acetylated 1-*O*-cholesteryl-β-d-glucopyranoside as a white solid (615 mg; 32% yield). Its structure was confirmed by comparison with a specimen previously reported in the literature (88).

Sodium methoxide (68 mg; 1.25 mmol) was added to a solution of acetylated 1-*O*-cholesteryl-β-d-glucopyranoside (600 mg; 0.84 mmol) in methanol (6 mL) and chloroform (6 mL). After 5 h at room temperature, the reaction mixture was treated with Dowex 50W-X8 (H^+^), filtered through a pad of Celite, and the solvent was evaporated. The crude product was purified by column chromatography [chloroform/methanol (20:1), (20:1.5), then (20:2)] followed by recrystallization with methanol to give 1-*O*-cholesteryl-β-d-glucopyranoside as a white solid (246 mg; 54% yield). mp 255-256 ºC (from MeOH) (Ref. 88); mp 265-267 ºC. RMN ^1^H (DMSO-*d*_6_, 400 MHz) δ = 5.33-5.34 (m, 1H), 4.89 (d, *J* = 4.7 Hz, 1H), 4.86 (d, *J* = 4.7 Hz, 2H), 4.42 (t, *J* = 5.8 Hz, 1H), 4.23 (d, *J* = 7.8 Hz, 1H), 3.65 (dd, *J* = 10.7, 4.9 Hz, 1H), 3.37-3.51 (m, 2H), 2.99-3.16 (m, 3H), 2.87-2.93 (m, 1H), 2.33-2.39 (m, 1H), 2.10-2.16 (m, 1H), 1.90-1.98 (m, 2H), 1.76-1.86 (m, 3H), 0.98-1.58 (m, 21H), 0.96 (s, 3H), 0.90 (d, *J* = 6.5 Hz, 3H), 0.86 (d, *J* = 2.1 Hz, 3H), 0.84 (d, *J* = 1.9 Hz, 3H), 0.66 (s, 3H).

Cell culture

J774.1 cells were grown in Dulbecco modified Eagle medium (DMEM Glutamax, Gibco, Thermo Fisher, Waltman, MA, USA), supplemented with 10% heat inactivated fetal bovine serum (FBS, Gibco), 1% sodium pyruvate (Gibco), 1% PenStrep (Gibco). Cells were maintained at 37 °C with 5% CO_2_ in a humidified incubator.

Activity-based probes





*Chemical Structure 2*: Chemical structures of GBA-specific ABP (ME569) and broad-spectrum glucosidase ABP (JJB367).

Hydrolase activity

Cathepsin-B, cathepsin-D, cathepsin-L and lysosomal acid lipase (LAL) activity were measured as previously described by us (3, 90) using 5 µg of protein per condition. GBA activity was assessed by activity-based profiling with ME569 as reported in (91) by running 30 µg of protein on a 10% SDS-PAGE gel followed by scanning for Cy5 fluorescence in a Chemidoc Touch Imaging System (Bio-Rad Laboratories). Bands were quantified using the Fiji software, v1.53f51.

Enzyme-linked Immunosorbent Assay (ELISA)

Supernatants from RAW cultures stimulated with lipids for 72 h were collected and stored at -20 ºC. The samples were analysed for TNF-α and IL-10 using the Mouse TNF-α Uncoated ELISA kit (Invitrogen, 88-7324) and Mouse IL-10 Uncoated ELISA kit (Invitrogen, 88-7105), according to manufacturer’s instructions. Samples (100 µL) were added to coated ELISA plate and incubated ON at 4 ºC. Synergy HT plate reader was employed to measure OD value at 450 nm, and the concentration of IL-6 was subsequently determined by the standard curve.

**Supplementary Table 1 –** List of primary antibodies used for immunofluorescence (IF) staining and western blotting (WB) in the main text.

| **Antibody** | **Company \| Catalogue number** | **Dilution** |
| --- | --- | --- |
| Rat anti-LAMP1 | DSHB \| 1D4B | IF: 1:500  WB: 1:500 |
| Rabbit anti-CTSD | Abcam \| ab75852 | WB: 1:1000 |
| Rabbit anti-p21 | Abcam \| ab109199 | IF: 1:100  WB: 1:1000 |
| Rabbit anti-phospho-TFEB (Ser211) | Cell Signaling \| 37681 | WB: 1:500 |
| Rabbit anti-TFEB | Bethyl Laboratories \| A303-673A | IF: 1:100  WB: 1:3000 |
| Rabbit anti-Lamin B1 | Abcam \| ab16048 | IF: 1:500  WB: 1:1000 |
| Mouse-anti phospho-Histone γH2A.X (Ser139) | Sigma \| 05-636 | IF: 1:500 |
| Rabbit anti-SQSTM1/ p62 | Abgent \| SA150305ZD | IF: 1:50 |
| Mouse anti-SQSTM1/ p62 | Abnova \| H00008878-M01 | WB: 1:500 |
| Rabbit anti-phospho-S6 Ribosomal Protein (Ser235/236) | Cell Signaling \| 4858S | WB: 1:2000 |
| Rabbit anti-S6 Ribosomal Protein | Cell Signaling \| 2217S | WB: 1:1000 |
| Rabbit anti-mTOR | Cell Signaling \| 2983S | WB: 1:1000 |
| Rabbit anti-phospho-mTOR (Ser2448) | Cell Signaling \| 5536S | IF: 1:100  WB: 1:500 |
| Rabbit anti-LDLR | Abcam \| ab52818 | WB: 1:1000 |
| Goat anti-gpNMB | R&D Biotechne \| AF2330 | WB: 1:500 |
| Rabbit anti-phospho-AMPK (Thr172) | Cell Signaling \| 2521S | WB: 1:1000 |
| Rabbit anti-AMPK | Cell Signaling \| 2532S | WB: 1:1000 |
| Rabbit anti-LC3B | Cell Signaling \| 2775S | WB: 1:1000 |
| Mouse anti-Tubulin | DSHB \| E7 | WB: 1:500 |
| Goat anti-GAPDH | SICGEN \| AB0067 | WB: 1:500 |
| Goat anti-Calnexin | SICGEN \| AB0041 | WB: 1:500 |

**Supplementary Table 2** - Antibodies used in the Supplementary Information for western blotting (WB).

| **Antibody** | **Company \| Catalogue number** | **Dilution** |
| --- | --- | --- |
| Rabbit anti-phospho STAT3 (Thy705) | Cell Signaling \| 9131S | WB: 1:1000 |
| Mouse anti-STAT3 (F-2) | Santa Cruz \| sc-8019 | WB: 1:1000 |
| Rabbit anti-TLR4 | Invitrogen \| 48-2300 | WB: 1:500 |
| Rabbit anti-phospho-NF-κB (Ser536) (93H1) | Cell Signaling \| #3033 | WB: 1:1000 |
| Rabbit anti-TNF-α | PeproTech \| 500-P64 | WB: 1:500 |
| Rabbit phospho-IκBα (Ser32) | Invitrogen \| MA5-15087 | WB: 1:500 |
| Rabbit IκBα | Invitrogen \| MA5-15153 | WB: 1:1000 |
| Mouse anti-p65 | DSHB \| PCRP-RELA-2B6-s | WB: 1:500 |

Quantitative RT-PCR

**Supplementary Table 3** **–** Primers sequences for RT-PCR used in the Supplementary Information.

| **Genes** | **Sequences** | | |
| --- | --- | --- | --- |
|  | **Forward** | **Reverse** |  |
| *Klf4* | GAAATTCGCCCGCTCCGATGA | CTGTGTGTTTGCGGTAGTGCC |  |
| *Cd11b* | AAACCACAGTCCCGCAGAGA | CGTGTTCACCAGCTGGCTTA |  |
| *Hmgr* | CCTTTGATAGCACCAGCAGATTT | AGCAGTGCTTTCTCCGTACC |  |
| *Lipa* | CTAGAATCTGCCAGCAAGCC | AGTATTCACCGAATCCCTCG |  |
| *Hmox* | CTAAGACCGCCTTCCTGCTC | GGGCAGTATCTTGCACCAGG |  |
| *Il10* | TGGCCCAGAAATCAAGGAGC | CAGCAGACTCAATACACACT |  |
| *Tnf* | GTCCCCAAAGGGATGAGAAGT | TTTGCTACGACGTGGGCTAC |  |
| *Pgk1* | ATGGATGAGGTGGTGAAAGC | CAGTGCTCACATGGCTGACT |  |
| *Gapdh* | GGGAAGCCCATCACCATCTTC | AGAGGGGCCATCCACAGTCT |  |

RT-qPCR with Taqman with the Taqman probes listed in the table below were carried out by using the Taqman Fast Advanced Master Mix (4444557, Applied Biosystems) according to manufacturer’s instructions.

**Supplementary Table 4** – Taqman probes used for RT-PCR in the Supplementary Information.

| **Gene** | **Catalogue number \| Dye** | **ID** |
| --- | --- | --- |
| *Npc1* | 4453320 \| FAM | Mm00435300_m1 |
| *Abca1* | 4453320 \| FAM | Mm00442646_m1 |
| *Gba* | 4453320 \| FAM | Mm00484700_m1 |
| *Gba2* | 4453320 \| FAM | Mm00554547_m1 |
| *Cd74* | 4453320 \| FAM | Mm00658576_m1 |

**Supplementary References**

88. Iga, D. P., S. Iga, R. R. Schmidt, and M. C. Buzas. 2005. Chemical synthesis of cholesteryl β-D-galactofuranoside and -pyranoside*. Carbohydr Res.* 340: 2052–2054.

89. Maslov, M. A., N. G. Morozova, E. I. Chizhik, D. A. Rapoport, E. I. Ryabchikova, M. A. Zenkova, and G. A. Serebrennikova. 2010. Synthesis and delivery activity of new cationic cholesteryl glucosides. *Carbohydr Res.* 345: 2438–2449.

90. Di Spiezio, A., A. R. A. Marques, L. Schmidt, N. Thießen, L. Gallwitz, J. Fogh, U. Bartsch, and P. Saftig. 2021. Analysis of cathepsin B and cathepsin L treatment to clear toxic lysosomal protein aggregates in neuronal ceroid lipofuscinosis. *Biochimica et Biophysica Acta (BBA) - Molecular Basis of Disease.* 1867: 166205.

91. Marques, A. R. A., J. Aten, R. Ottenhoff, van R. C. P, H. M. D, N. Claessen, V. V. M. F, K. Zhou, Z. Lin, M. Mirzaian, R. G. Boot, D. Z. C. I, H. S. Overkleeft, Y. Yildiz, J. M. F. G. Aerts, C. P. A. A. van Roomen, D. Herrera Moro, N. Claessen, M. F. Vinueza Veloz, K. Zhou, Z. Lin, M. Mirzaian, R. G. Boot, C. I. De Zeeuw, H. S. Overkleeft, Y. Yildiz, and J. M. F. G. Aerts. 2015. Reducing GBA2 activity ameliorates neuropathology in Niemann-Pick type C mice. *PLoS One.* 10: e0135889.

**Supplementary Figure Legends**

**Supplementary Figure 1 – Glycosphingolipids and lysosomal hydrolase activity in the core of human atherosclerotic lesions.** Lipid levels in human CAE specimens and control (surrounding) tissue determined by LC-MS/MS. Levels of the glycosphingolipid globotriaosylceramide (Gb3) **(A)** (namole per gram of wet weight) in human CAE samples. Lyso-glycosphingolipids sphinganine **(B)**, lyso-sphingomyelin **(C)**, Lyso-Gb3 **(D)** and lactosylsphingosine (LacSph) **(E)** (picomole per gram of wet weight) in CAE samples. **(F)** Enzymatic activity of the lysosomal hydrolases cathepsin-B (CTSB), cathepsin-L (CTSL), cathepsin-D (CTSD), lysosomal acid lipase (LAL) and glucocerebrosidase (GBA) in CAE samples and surrounding tissue (control, Ct). Data = violin blot of 6-11 samples. The *p* values were obtained by Mann-Whitney test; ns = nonsignificant; **p* < 0.05.

**Supplemental Figure 2** **– Glysosphingolipids and lysoglycosphingolipids in the plasma of patients suffering from CVDs. (A)** Hexosyl-ceramide (nanomole per millilitre), **(B)** ceramide (nanomole per millilitre), **(C)** dihydroceramide (nanomole per millilitre), **(D)** hexosyl-sphingosine (picomole per millilitre) and **(E)** lyso-Gb3 (picomole per millilitre) in the plasma of patients that suffered from ischemic stroke, angina pectoris and myocardial infarction, and healthy age- and gender-matched individuals (N=22-40). Patients were divided according to the prescription or not of statins at the date of sample collection. Data mean ± SD analysed by Kruskal-Wallis followed by Dunn’s multiple comparison test: **p*<0.05, ***p*<0.01, ****p*<0.001.

**Supplementary Figure 3 – Vascular smooth muscle cell-derived foam cells do not accumulate GlcChol. (A)** GlcCer levels (picomole per milligram per protein) determined by LC-MS/MS in the lysates of RAW cells treated with 807 µM of POPC or 1500 µM of ChA for 72 h. Data = mean ± SD of 6 independent experiments; **p* < 0.05 (unpaired t-test). **(B)** Total cholesterol levels (micromole per milligram of protein) determined by fluorometric methodology in the lysates of MOVAS cells exposed to 807 µM POPC or 1500 µM ChA for 72 h. GlcCer **(C)** and GlcChol **(D)** levels (picomole per milligram of protein) determined by LC-MS/MS in the lysates of lysates of MOVAS cells exposed to 807 µM POPC or 1500 µM ChA for 72 h. Data = mean ± SD of 3 independent experiments; ns = nonsignificant; **p* < 0.05 (unpaired t-test). **(E)** Fluorescent labelling of active GBA with the ABP ME569 and GBA2 with the ABP JJB367 in lysates of cells exposed to POPC or ChA for 72 h in simultaneous with CBE (GBA inhibitor), MZ21 (GBA2 inhibitor), cyclodextrin (CD) (lysosomal cholesterol exporter), Eliglustat (GCS inhibitor) or Lalistat 2 (LAL inhibitor). Gels were stained with Coomassie Brilliant Blue (CBB) as loading control. Heatmaps representing Globotriaosylceramide (Gb3) **(F)** and Lactosylceramide (LacCer) **(G)** levels (picomole per milligram of protein) in RAW 264.7 cells exposed to POPC or ChA for 72 h in simultaneous with CBE, MZ-21, cyclodextrin (CD), Eliglustat or Lalistat 2. Data = mean ± SD of 3-12 independent experiments, **p* < 0.05, *****p* < 0.0001 (ANOVA-Tukey test relative to control POPC).

**Supplementary Figure 4 – Protein levels and organelle morphology in Mφ exposed to GlcChol.** **(A)** Percentage of cells in each phase of the cell cycle following exposure to POPC, Chol or GlcChol for 72 h determined by PI flow cytometry analysis. **(B)** Representation of the gating strategy preformed in FlowJo™ v10.10 software, using the Watson pragmatic fitting algorithm for cell cycle analysis. **(C)** Representation of the gating strategy preformed in FlowJo™ v10.10 software for apoptosis analysis. mRNA expression levels of *Hmox1* **(D)**, *Klf4* **(E)**, *Cd11b* **(F)**, *Cd74* **(G)**, *Il10* **(H)** and *Tnf* **(I)** in RAW 264.7 cells exposed to POPC (54 µM), Chol (100 µM) and GlcChol (100 µM) for 72 h. mRNA levels were assessed by qRT-PCR. Data were normalized to the endogenous *Gapdh* and *Pgk1* genes. The values are mean ± SD of 4-5 independent experiments. The *p* values were obtained paired t-test; **p* < 0.05; ***p* < 0.01. TNF-α **(J)** and IL-10 **(K)** levels (picogram per milligram of cellular protein) in the media of Mφ treated with liposomes for 72 h. Cytokine levels were assessed by ELISA assay. LPS (1000 ng/mL) stimulation was used as positive control. Data = mean ± SD of 3 independent experiments; ns = nonsignificant; **p* < 0.05; ***p* < 0.01; (Two-way ANOVA-Tukey test). Immunoblots **(L)** and densiometric quantification of the protein levels of TLR4 **(M)**, phospho-NFκB **(N)**, NFκB **(O)**, phosphorylated/total NFκB ratio **(P)**, phospho-IκBα **(Q)**, IκBα **(R)**, phosphorylated/total IκBα ratio **(S)**, TNF-α **(T)**, phospho-STAT3 **(U)**, STAT3 **(V)** and the phosphorylated/total STAT3 ratio **(X)** in lysates of cells treated with POPC, Chol or GlcChol for 72 h. Protein levels were normalized to calnexin (CANX) or GAPDH levels. Data = mean ± SD of 3 independent experiments; ns = nonsignificant; **p* < 0.05; ***p* < 0.01; ****p* < 0.001 (ANOVA-Tukey test).

**Supplementary Figure 5 – Protein levels and organelle morphology in Mφ exposed to GlcChol.** mRNA expression levels of *Hmgr* **(A)**, *Lipa* **(B)**, *Abca1* **(C)**, *Npc1* **(D)**, *Gba* **(E)** and *Gba2* **(F)** in RAW 264.7 cells exposed to POPC (54 µM), Chol (100 µM) and GlcChol (100 µM) for 72 h. mRNA levels were assessed by qRT-PCR. Data were normalized to the endogenous *Gapdh* and *Pgk1* genes. The values are mean ± SD of 4-5 independent experiments. The *p* values were obtained paired t-test; **p* < 0.05; ***p* < 0.01. Immunoblots **(G)** and densiometric quantification of the protein levels of PEX26 **(H)**, citrate synthase **(I)**, TOMM20 **(J)**, ATP5A (Complex V of the OXPHOS) **(K)**, MTOC1 (Complex IV) **(L)**, UQCRC2 (Complex III) **(M)**, SDHB (Complex II) **(N)**, NDUFB8 (Complex I) **(O)**, and NPC1 **(P)** in lysates of cells treated with POPC, Chol or GlcChol for 72 h. Protein levels were normalized to calnexin (CANX) or GAPDH levels. Data = mean ± SD of 3-8 independent experiments; ns = nonsignificant; **p* < 0.05 (ANOVA-Tukey test). **(Q)** Representative images of EEA1, GM130 and TOMM20 in RAW 264.7 Mφ incubated with 100 µM GlcChol or with control liposomes for 72 h. Nuclei were labelled with DAPI. Early-endosomes are coloured magenta, Golgi is green, and mitochondria are cyan. Confocal single-slice images. The insets are enlargements of the areas outlined with the white boxes. Scale bars, 10 and 2 μm in the insets.

**Supplementary Figure 6 – Impact of GlcChol on the organelles of J774.1 murine Mφ. (A)** Representative images of Phalloidin, GM130 and TOMM20 in J774.1 murine Mφ incubated with 100 µM GlcChol or with control liposomes for 72 h. Nuclei were labelled with DAPI. F-actin is coloured magenta, Golgi is green, and mitochondria are cyan. Confocal single-slice images. The insets are enlargements of the areas outlined with the white boxes. Scale bars, 10 and 2 μm in the insets. **(B)** Representative images of PLIN2, BODIPY FL and LAMP1 in J744.1 Mφ incubated with 100 µM GlcChol or with control liposomes for 72 h. Nuclei were labelled with DAPI. Lysosomes are coloured magenta, neutral lipids are green, and lipid droplets are cyan. Confocal single-slice images. The insets are enlargements of the areas outlined with the white boxes. Scale bars, 10 and 2 μm in the insets. Immunoblots **(C)** and densiometric quantification of the protein levels of LAMP1 **(D)**, CTSD **(E)**, mature/total CTSD ratio **(F)** and p21 **(G)** in lysates of cells treated with POPC, Chol or GlcChol for 72 h. Protein levels were normalized to calnexin (CANX) levels. Data = mean ± SD of 6 independent experiments; **p* < 0.05; ***p* < 0.01 (Two-way ANOVA-Tukey test). Cytokine IL-6 **(H)** and IL-1β **(P)** levels (picogram per milligram of cellular protein) in the media of Mφ pre-conditioned with liposomes for 24 h and then exposed to LPS for 24 h. Cytokine and chemokine levels were assessed by flow cytometry multiplex assay. Data = mean ± SD of 3-5 independent experiments; ns = nonsignificant (Two-way ANOVA-Tukey test).

**Supplementary Figures**

**Supplementary Figure 1**


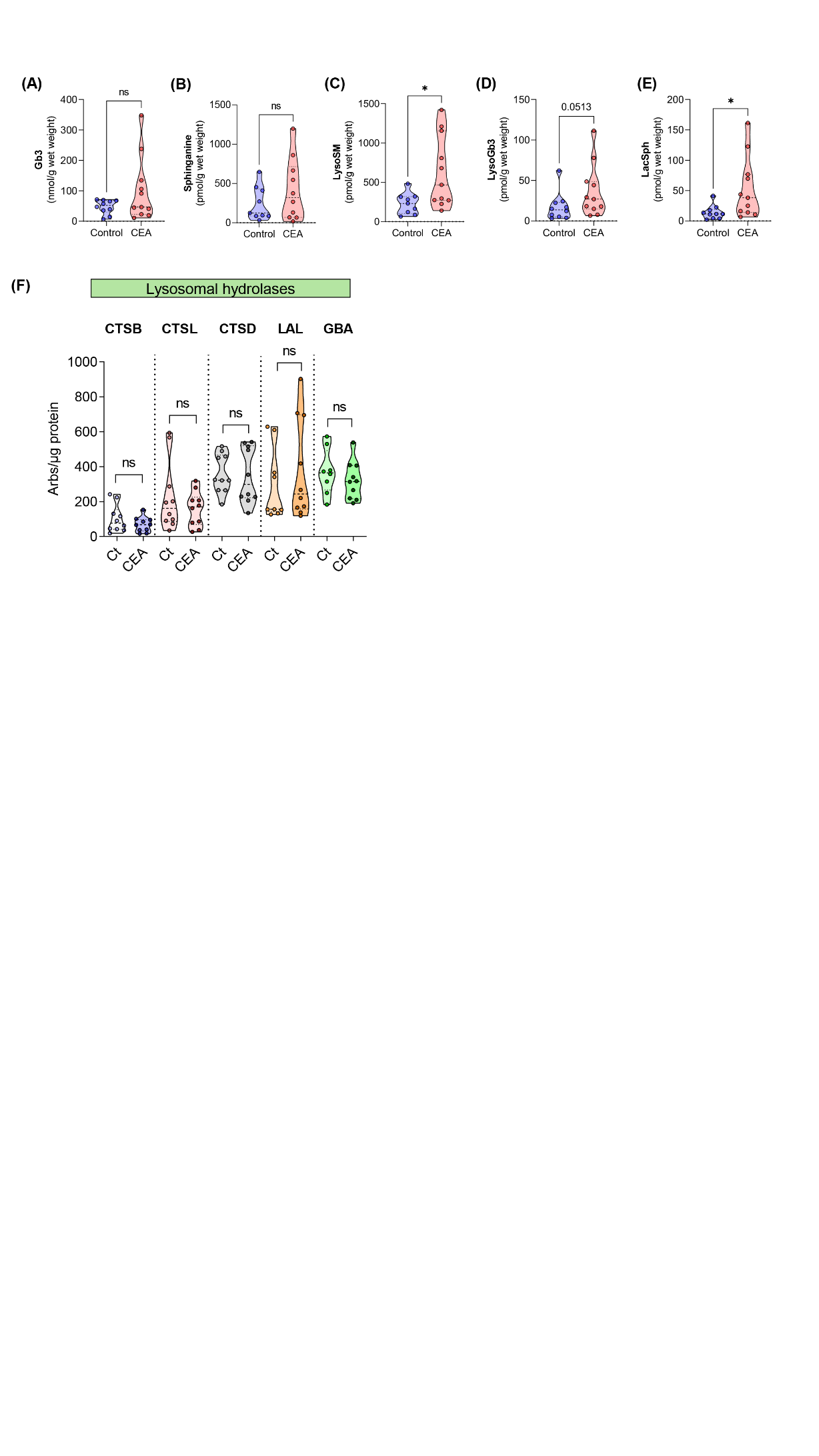


**Supplementary Figure 2**


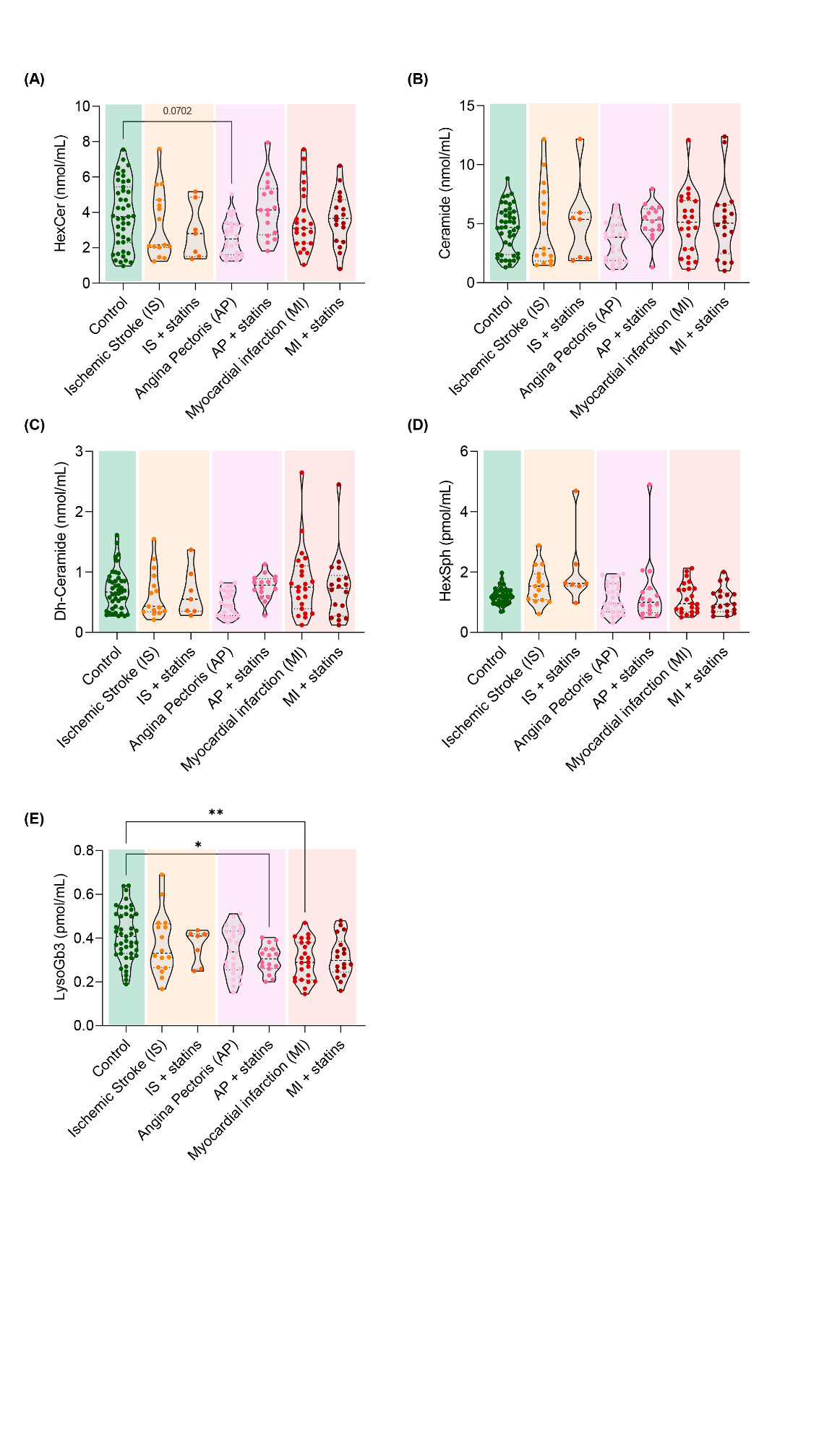


**Supplementary Figure 3**


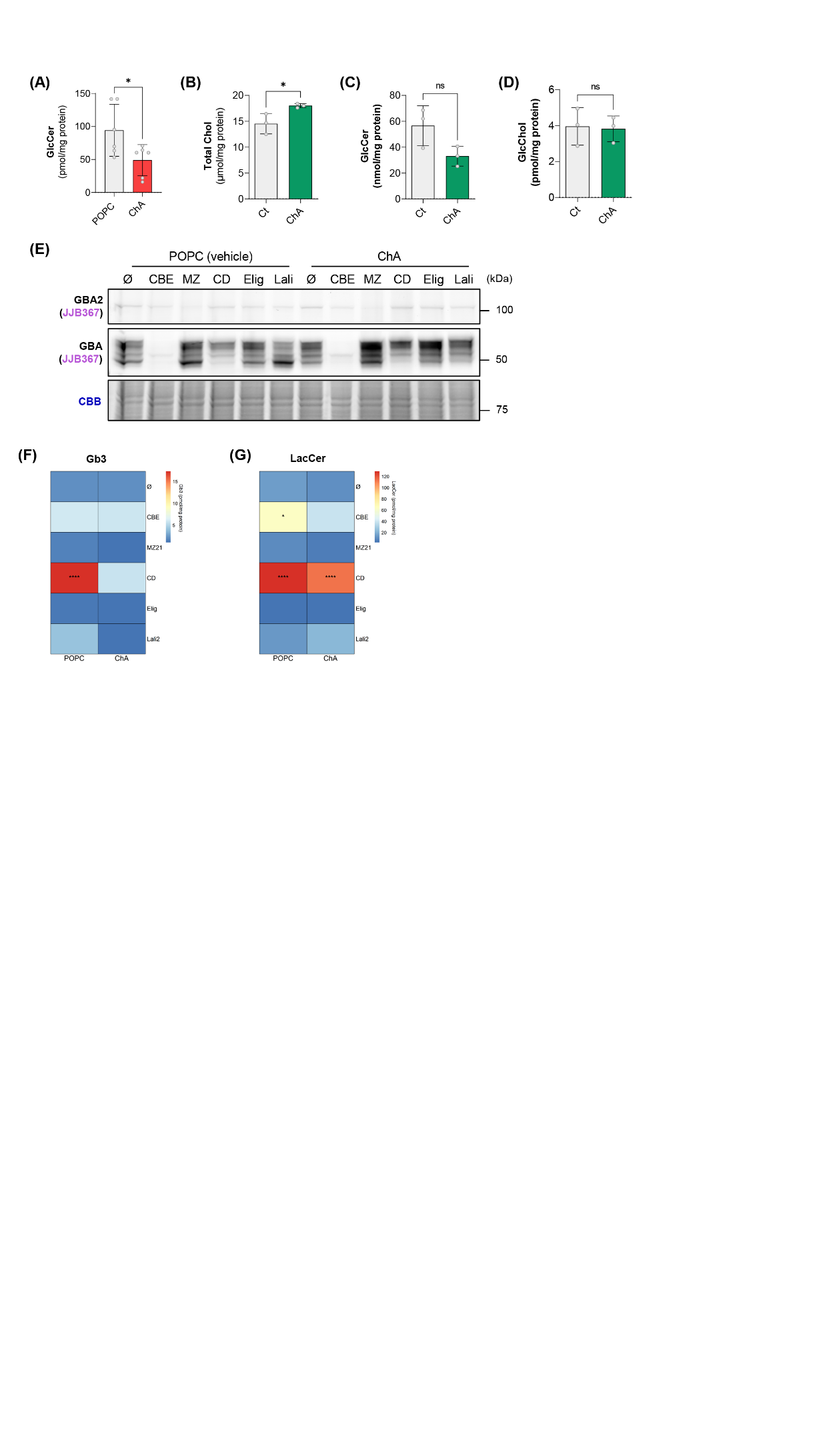


**Supplementary Figure 4**


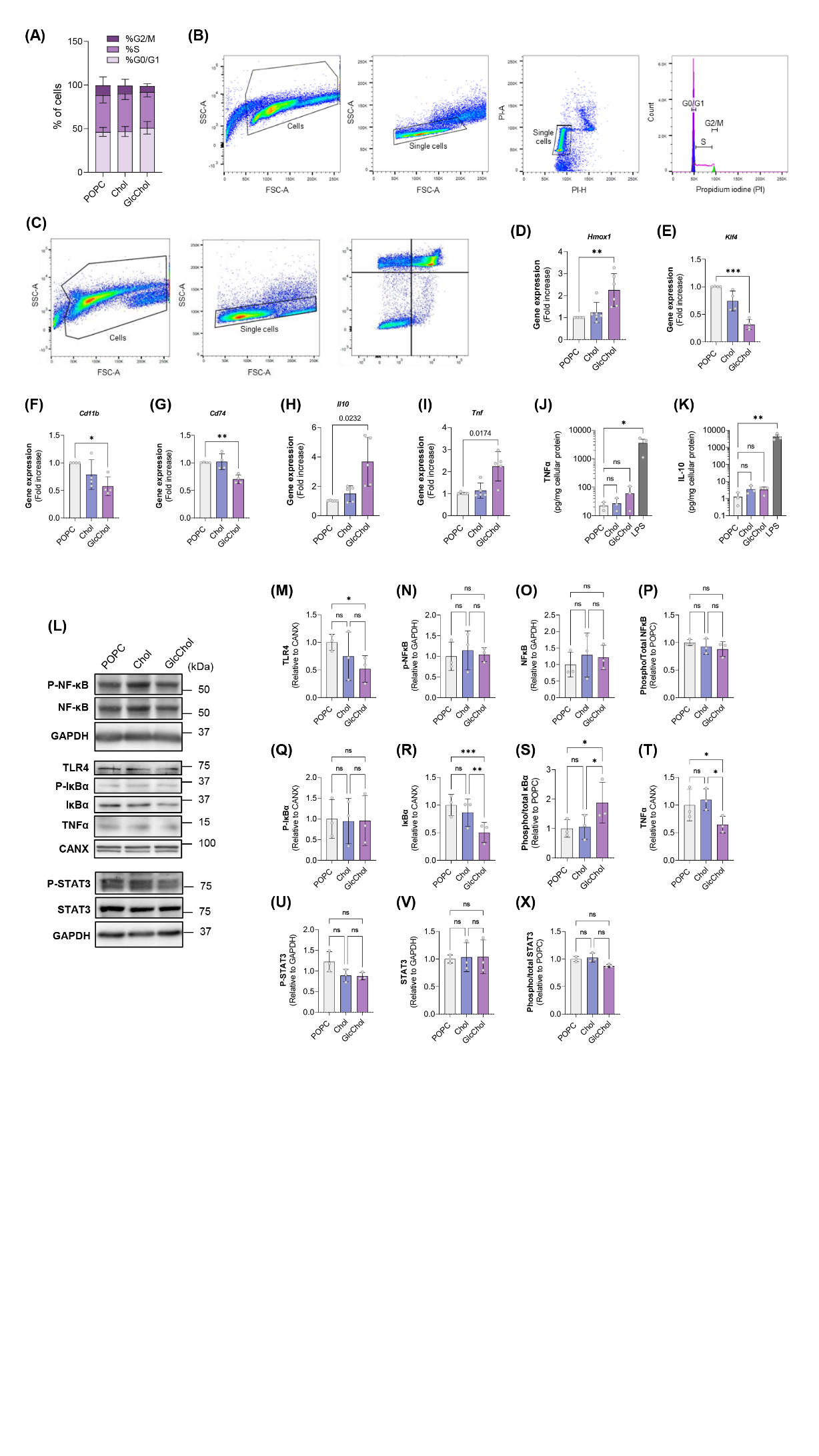


**Supplementary Figure 5**


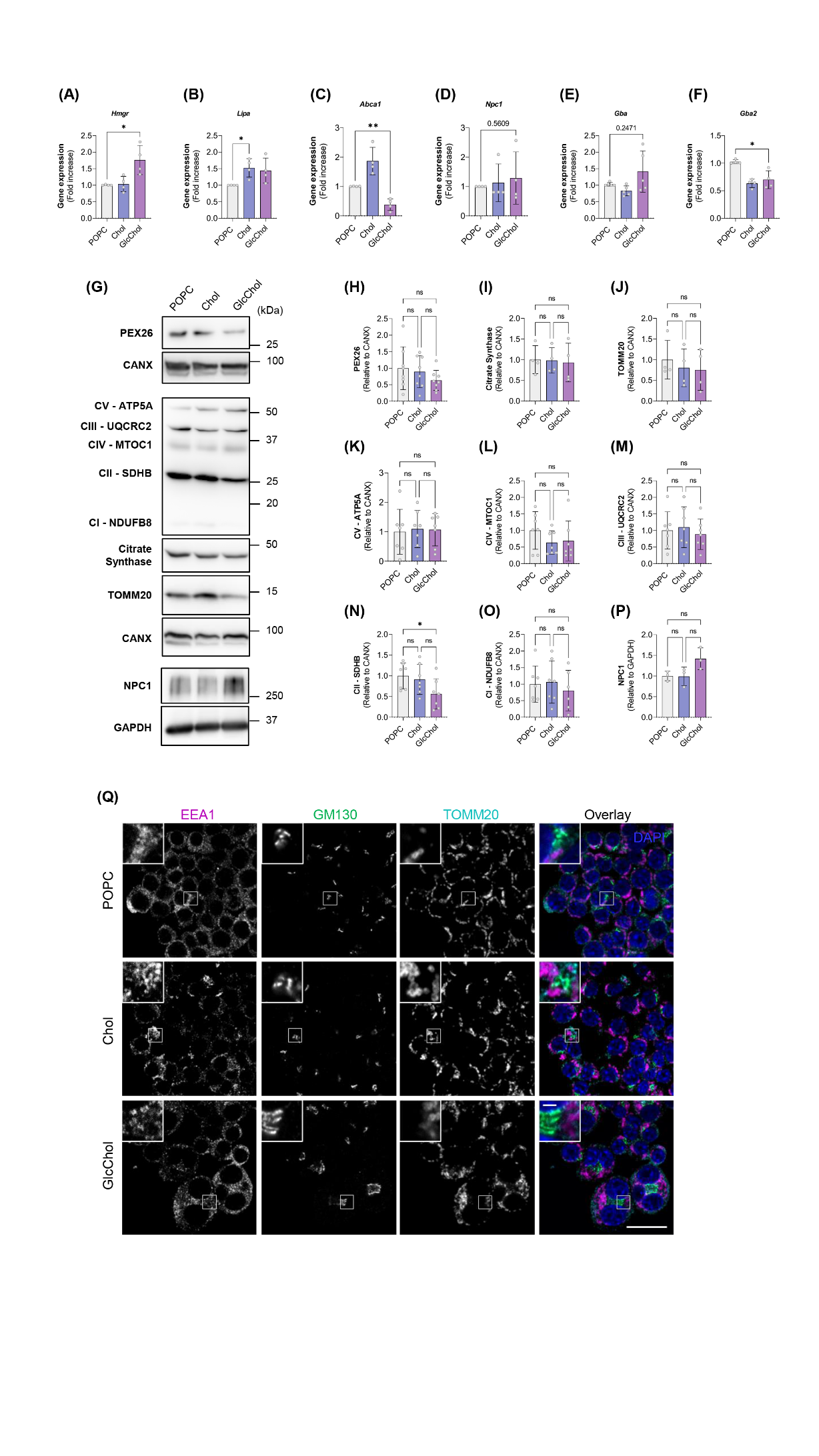


**Supplementary Figure 6**


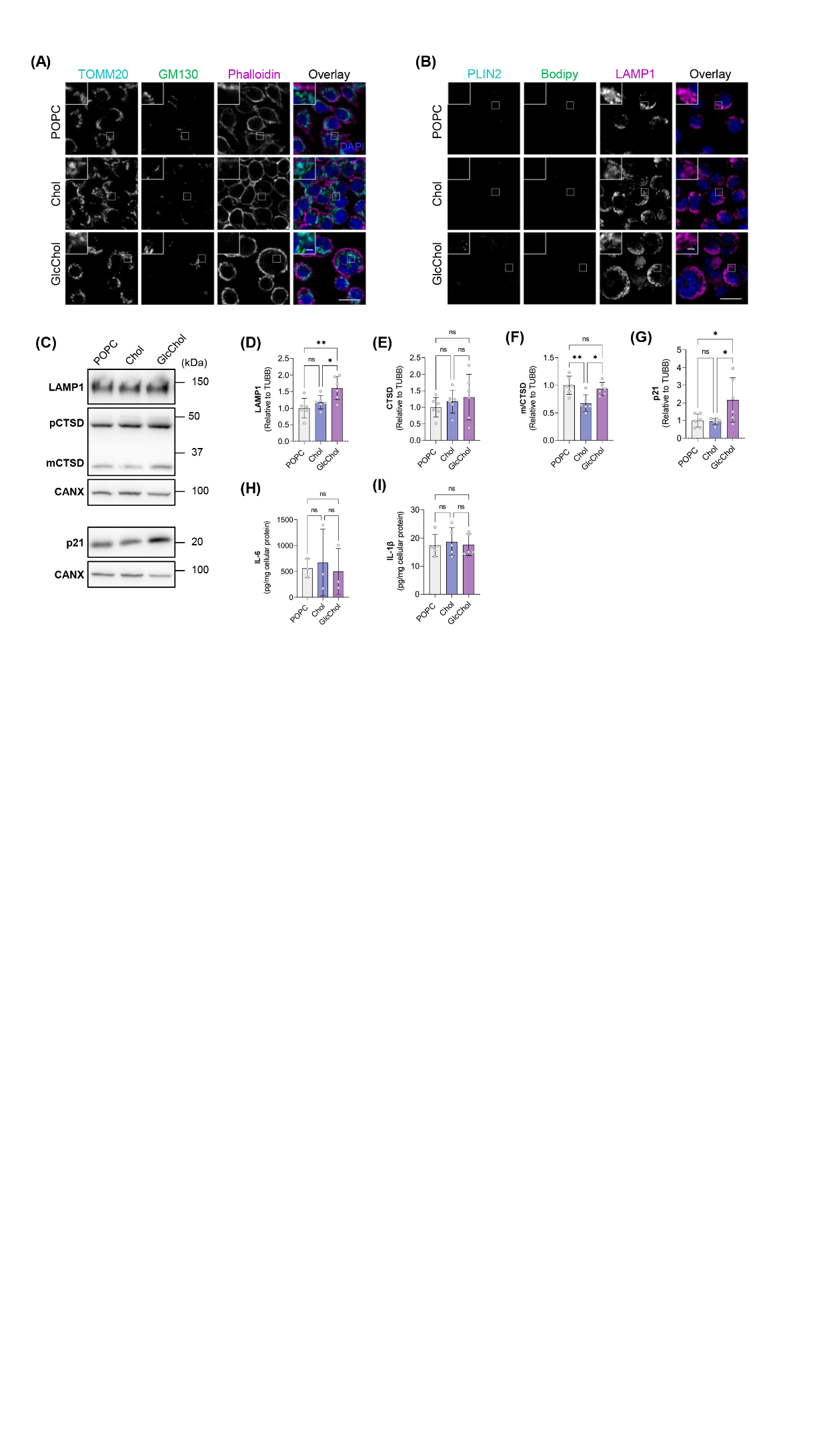

Supplement: Supplementary information [file mmc1.docx]
